# Bilateral gene interaction hierarchy analysis of the cell death gene response emphasizes the significance of cell cycle genes following unilateral traumatic brain injury

**DOI:** 10.1186/s12864-016-2412-0

**Published:** 2016-02-24

**Authors:** Todd E. White, Monique C. Surles-Zeigler, Gregory D. Ford, Alicia S. Gates, Benem Davids, Timothy Distel, Michelle C. LaPlaca, Byron D. Ford

**Affiliations:** Department of Neurobiology, Neuroscience Institute, Morehouse School of Medicine, 720 Westview Drive SW, Atlanta, GA 30310 USA; Division of Natural Sciences and Physical Education, Georgia Highlands College, 5441 Highway 20, NE, Cartersville, GA 30121 USA; Department of Biomedical Engineering, Georgia Institute of Technology, 313 Ferst Drive, Atlanta, GA 30332 USA; University of California-Riverside School of Medicine, 900 University Ave., Riverside, CA 92521 USA

**Keywords:** Traumatic brain injury, Cell death, Microarray, Bioinformatics, Gene interaction hierarchy

## Abstract

**Background:**

Delayed or secondary cell death that is caused by a cascade of cellular and molecular processes initiated by traumatic brain injury (TBI) may be reduced or prevented if an effective neuroprotective strategy is employed. Microarray and subsequent bioinformatic analyses were used to determine which genes, pathways and networks were significantly altered 24 h after unilateral TBI in the rat. Ipsilateral hemi-brain, the corresponding contralateral hemi-brain, and naïve (control) brain tissue were used for microarray analysis.

**Results:**

Ingenuity Pathway Analysis showed cell death and survival (CD) to be a top molecular and cellular function associated with TBI on both sides of the brain. One major finding was that the overall gene expression pattern suggested an increase in CD genes in ipsilateral brain tissue and suppression of CD genes contralateral to the injury which may indicate an endogenous protective mechanism. We created networks of genes of interest (GOI) and ranked the genes by the number of direct connections each had in the GOI networks, creating gene interaction hierarchies (GIHs). Cell cycle was determined from the resultant GIHs to be a significant molecular and cellular function in post-TBI CD gene response.

**Conclusions:**

Cell cycle and apoptosis signalling genes that were highly ranked in the GIHs and exhibited either the inverse ipsilateral/contralateral expression pattern or contralateral suppression were identified and included STAT3, CCND1, CCND2, and BAX. Additional exploration into the remote suppression of CD genes may provide insight into neuroprotective mechanisms that could be used to develop therapies to prevent cell death following TBI.

**Electronic supplementary material:**

The online version of this article (doi:10.1186/s12864-016-2412-0) contains supplementary material, which is available to authorized users.

## Background

Traumatic brain injury (TBI) is a major public health problem in both the civilian and military populations as TBI has now become a prominent injury in war zones. Of the 1.7 million new TBIs that are sustained annually in the United States [1], 53,000 result in death [2] while an additional 125,000 leave the affected people with long-term behavioral deficits [3]. Overall, about 3 million Americans are currently suffering with chronic effects of TBI [4]. Additionally, it is estimated that 17–30 % of soldiers returning for Iraq and Afghanistan have suffered TBIs [5, 6]. Development of more effective clinical treatments is necessary to reduce the healthcare and financial burden of TBI. Such development requires basic experimentation into the mechanisms underlying TBI.

Primary damage to cells by TBI may be irreversible and lead to immediate cell death, however, delayed or secondary cell death that is caused by a cascade of cellular and molecular processes initiated by the trauma [7–10] may be reduced or prevented if an effective neuroprotective strategy is employed. Development of such a strategy requires an understanding of the molecular environment in the injured brain so that deleterious molecules and processes can be identified and inhibited. A step towards understanding the molecular response to TBI is examining gene expression profiles following the injury.

Microarray technology allows for examination of thousands of genes in one assay. The key to using this technology is interpreting the resulting gene expression patterns and using the interpreted data to guide further study. The development of advanced bioinformatic analysis tools have aided in deciphering microarray data. One such tool is the Ingenuity Pathway Analysis (IPA) software program which uses a database built from published scientific literature to draw direct and indirect interactions between genes and to assign genes to specific biological functions, canonical pathways, and networks [11]. IPA also features a strong network building component that allows for the creation and analysis of networks composed of any genes of interest (GOI). We have previously devised a method for using the initial information that IPA provides and subsequent network analysis to determine which genes are most significant to the inflammatory response following neuronal injury unilateral controlled cortical impact (CCI) in the rat [12]. This analysis results in a gene interaction hierarchy (GIH) where genes of interest are ranked based on the number of interactions they have with each other. The theory behind the analysis is that a gene that interacts with more genes in a particular set of genes has the potential to influence that set of genes the most.

The current study uses gene expression profiling and bioinformatic analysis to examine the cell death gene response 24 h following unilateral CCI. One significant finding of our previous study was that while inflammatory gene expression was induced on the ipsilateral side of the brain following TBI, there was a suppression of inflammatory genes contralateral to the injury [12]. We believe that this endogenous anti-inflammatory response may hold clues for the development of anti-inflammatory treatments for TBI and other acute brain injuries. Inflammation resulting from many different types of acute brain injuries, including TBI and ischemic stroke, has been linked to subsequent neuronal cell death [13–16]. By extension, we believe that understanding the post-TBI expression of genes involved in acute cell death will provide clues for the development of neuroprotective strategies.

## Methods

### Animals

All animals used in these studies were treated humanely and with regard for alleviation of suffering and pain and all protocols involving animals were approved by the IACUCs of Morehouse School of Medicine and/or The Georgia Institute of Technology prior to the initiation of experimentation. Adult male Sprague–Dawley rats (290–300 g; Charles River Laboratories International, Inc., USA) were housed individually in standard plastic cages in a temperature-controlled room (22 ± 2 °C) on a 12 h reverse light–dark cycle. Food and water were provided ad libitum.

### Controlled cortical impact

Under isoflurane anesthesia, rats received a unilateral controlled cortical impact (CCI/TBI) using the Pittsburgh Precision Instruments, Inc. device. A craniotomy was made with the center 4 mm posterior and 3–4 mm lateral to bregma using a 6 mm diameter trephan drill bit. The impact was done at an angle of 15° from vertical with a velocity of 3 m/s to a depth of 2 mm using a 5 mm diameter impact tip. These parameters were chosen to produce a moderate injury [17]. The rats were sacrificed 24 h post-injury and the brains were removed for RNA isolation or histology.

### RNA preparation and GeneChip analysis

The ipsilateral hemi-brain tissue at the site of the injury, the corresponding contralateral hemi-brain tissue, and naïve (control) brain tissue (*n =* 3 for each) were used for RNA isolation. Total RNA was extracted with TRIzol Reagent (Life Technologies, Rockville, MD, USA) and cleaned (RNAqueous Kit, Ambion, Austin, TX, USA). The RNA was prepared for microarray hybridization with the GeneChip® 3′ IVT Express Kit (Affymetrix Inc., Santa Clara, CA, USA) aRNA amplification procedure. Briefly, total RNA was reverse transcribed to synthesize first-strand cDNA containing a T7 promoter sequence. The single-stranded cDNA was converted into a double-stranded DNA template for transcription. The reaction employed DNA polymerase and RNase H to simultaneously degrade the RNA and synthesize second-strand cDNA. *In vitro* transcription generated multiple copies of biotin-modified aRNA from the double-stranded cDNA templates (this was the amplification step). aRNA Purification removed unincorporated NTPs, salts, enzymes, and inorganic phosphate to improve the stability of the biotin-modified aRNA. Finally, the labeled aRNA was fragmented to prepare the sample for hybridization to GeneChip® 3′ expression arrays [18]. Following fragmentation, 15 μg of the biotinylated cRNA was hybridized to an Affymetrix Rat Genome 230 2.0 GeneChip. The chips were hybridized at 45 °C for 16 h, and then washed, stained with streptavidin–phycoerythrin and scanned according to manufacturing guidelines.

### Microarray data analysis

Data analysis was performed using Affymetrix Expression Console™ software that supports probe set summarization and CHP file generation of 3′ expression using the MAS5 Statistical algorithm. Affymetrix microarrays contain the hybridization, labeling and housekeeping controls that help determine the success of the hybridizations. The Affymetrix Expression Analysis algorithm uses the Tukey’s biweight estimator to provide a robust mean Signal value and the Wilcoxon’s rank test to calculate a significance or p-value and Detection call (present, marginal or absent) for each probe set. The Detection p-value is calculated using a Discrimination Score [R] for all probes. The Discrimination Score is a basic property of a probe pair that describes its ability to detect its intended target. It measures the target-specific intensity differences of the probe pair (perfect match (PM) – mismatch (MM)) relative to its overall hybridization intensity (PM + MM). Background estimation is provided by a weighted average of the lowest 2 % of the feature intensities. Mismatch probes are utilized to adjust the perfect match (PM) intensity. Linear scaling of the feature level intensity values, using the trimmed mean, is the default to make the means equal for all arrays being analyzed. False-negative and false-positive rates are minimized by subtracting nonspecific signal from the PM probe intensities and performing an intensity-dependent normalization at the probe set level. Three chips were used for each experimental group: ipsilateral, contralateral and naïve control. The dataset produced by the Affymetrix software contains gene identifiers, corresponding expression values, and determination of whether genes are confirmed as present, marginal or absent. Previous principle component analysis of the raw datasets demonstrated that ipsilateral, contralateral and naïve clustered together by injury status and each group was well isolated from the other two groups [12]. The data were analyzed in Microsoft Excel for calculation of fold change and whether the genes were confirmed as present in the tissue sample. Genes in the injured brain that increased or decreased in expression by 2-fold or more compared to controls and were present in either all 3 ipsilateral samples or all 3 contralateral samples were identified. The gene datasets that were generated were ipsilateral vs. naïve (TBI-I) and contralateral vs. naïve (TBI-C) fold changes.

### Ingenuity pathway analysis

The gene datasets were analyzed between December 3, 2014 and January 8, 2015 using Ingenuity Pathway Analysis (Ingenuity® Systems, www.ingenuity.com) and overlaid onto a global molecular network developed from information contained in the Ingenuity Knowledge Base. The right-tailed Fisher’s Exact Test was used to determine the likelihood that the association between a set of experimental genes and a given biological function or pathway is not due to random chance [19]. In general, p-values less than 0.05 indicate a statistically significant, non-random association. The functions, canonical pathways, and gene networks that were most significant to the dataset were identified. Gene expression profiles were overlaid on the canonical pathway and gene network figures to reveal similarities and dissimilarities in their gene expression patterns. Gene networks were also created using Ingenuity Knowledge Base to further understand specific interactions between our genes of interest.

### TBI-I/TBI-C ratio

We used the following formulas to calculate the ratio of TBI-I to TBI-C fold changes: (1) Gene increased on both sides (TBI-I > TBI-C): ratio = (TBI-I)/(TBI-C); (2) Gene decreased on both sides (TBI-I > TBI-C): ratio = 1/[(TBI-I)/(TBI-C)]; (3) Gene decreased on both sides (TBI-I < TBI-C): ratio = −1/[(TBI-C)/(TBI-I)]; (4) Gene increased ipsilaterally and decreased contralaterally: ratio = (TBI-I)/-[1/(TBI-C)]; (5) Gene decreased ipsilaterally and increased contralaterally: ratio = (TBI-C)/[1/(TBI-I)].

### Histology

At 24 h post injury, rats were anesthetized with an intraperitoneal injection of a ketamine:xylazine:acetylpromazine cocktail (50:10:1.67 mg/kg respectively) and perfused transcardially with saline followed by cold 4 % paraformaldehyde solution in PBS for 30 min. Brains were quickly removed and cryoprotected in 30 % sucrose. The brains were then frozen in OCT mounting medium and stored until sectioning. Coronal sections of 20 μm thickness were cryosectioned from the perilesional brain area of each animal. Sections were mounted on slides which were stored at −80 °C until further processed. Fluoro-Jade® B (AG310, Millipore, Billerica, MA) labeling was performed as previously described [20]. TUNEL staining was performed using the TUNEL reaction mixture from the In Situ Cell Death Detection Kit, TMR red (12 156 792 910, Roche Diagnostics, Mannheim, Germany). Briefly, slide-mounted sections were post-fixed with 4 % paraformaldehyde for 15 min followed by a 10 min incubation in a 20 μg/mL proteinase K solution in 100 mM Tris HCl (pH 8.0) and 50 mM EDTA. The sections were then incubated for 60 min at 37 °C in the TUNEL reaction mixture. Phosphate buffered saline was used to rinse the sections after each step. A Zeiss fluorescence microscope equipped with a CCD camera (Carl Zeiss Microimaging, Inc., Thornwood, NY) was used to capture digital images of the sections.

### Real-time polymerase chain reaction (PCR)

RNA was extracted as above and quantified using the Nanodrop 2000c (Thermo Scientific, Waltham, MA). Equal amounts of ipsilateral, contralateral, and naïve RNA (*n =* 2 for each) were converted to cDNA using the iScript™ Reverse Transcription Supermix for RT-qPCR (170–8840, Bio-Rad Laboratories, Inc., Hercules, CA). The resulting product was diluted 1:100 with RNase-free sterile water. The diluted product was used in the real-time PCR analysis using the Quantitect SYBR® Green PCR Kit (204143, Qiagen, Hilden, Germany), custom oligo primers for SPP1, HSPB1, STAT3, CCND1, and GAPDH (reference gene) (Life Technologies, Rockville, MD), and a Bio-Rad CFX96™ Real-Time System mounted on a C1000™ Thermal Cycler. All steps were carried out according to manufacturer’s protocols. The real-time PCR results were analyzed using the ΔΔCt method where ΔCt1 = Ct (Target A‐exp) – Ct (GAPDH-exp); ΔCt2 = Ct (Target A‐naïve) –Ct (GAPDH‐naïve); and ΔΔCt = ΔCt1 – ΔCt2. The normalized target gene expression level was given by 2^-ΔΔCt^. The results were compared pairwise using a one-tail *T*-test assuming equal variance. Differences were considered significant when *p <* 0.05.

## Results

### Functional analysis

**Fig. 1:**
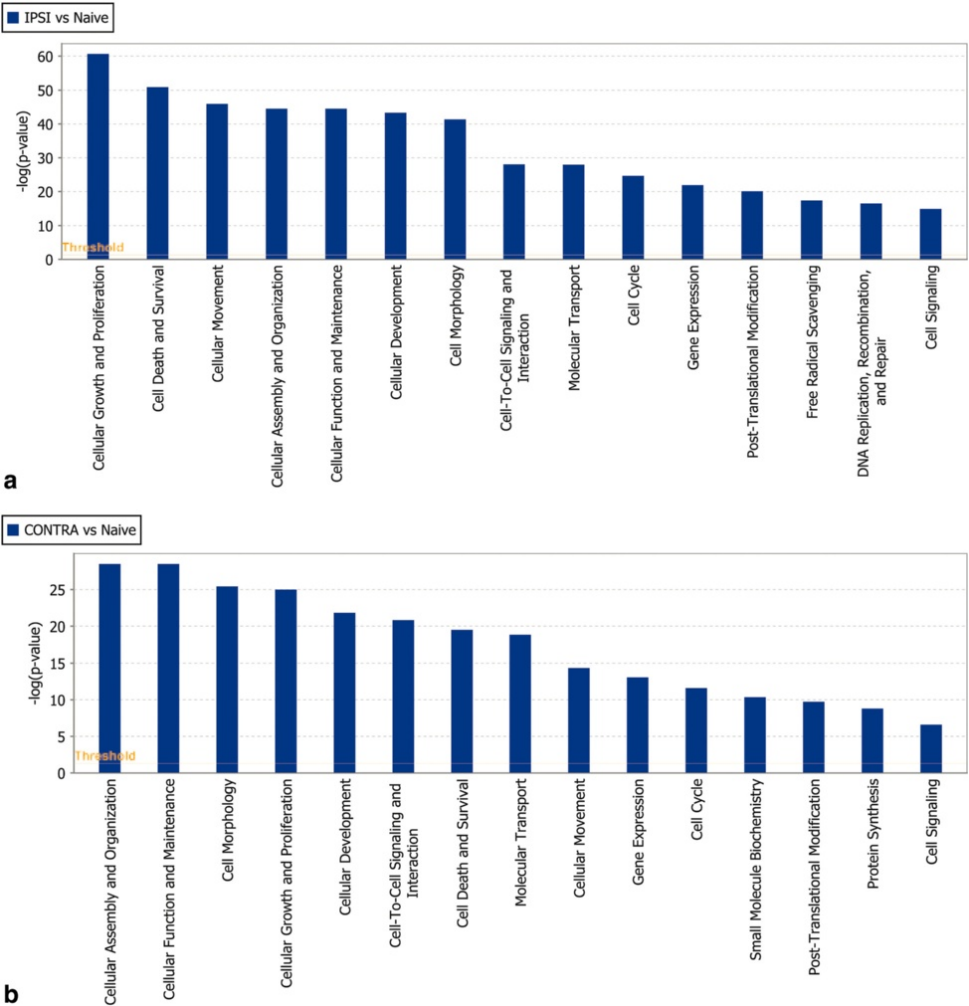
Overall functional analysis. Analysis of the top 15 molecular and cellular functions determined by IPA for the TBI-I (ipsilateral vs. naïve) dataset (**a**) and the TBI-C (contralateral vs. naïve) dataset (**b**) showed that cell death and survival was a top ranked function on both sides of the brain

To begin understanding the cell death gene response following TBI, we first looked at the biological functions associated with our datasets. Analysis of the top 15 molecular and cellular functions associated with the TBI-I (ipsilateral vs. naïve) and TBI-C (contralateral vs. naïve) datasets in IPA showed that cell death and survival (CD) was the second ranked TBI-I function that is also ranked in the top 7 functions for TBI-C (Fig. 1a, b). Also ranked in the top 7 molecular and cellular functions for both datasets are cellular growth and proliferation, cellular assembly and organization, cellular function and maintenance, cellular development, and cell morphology. Cellular movement and cell-to-cell signaling and interaction are ranked in the top 7 only for TBI-I and TBI-C, respectively.

### Histology

**Fig. 2:**
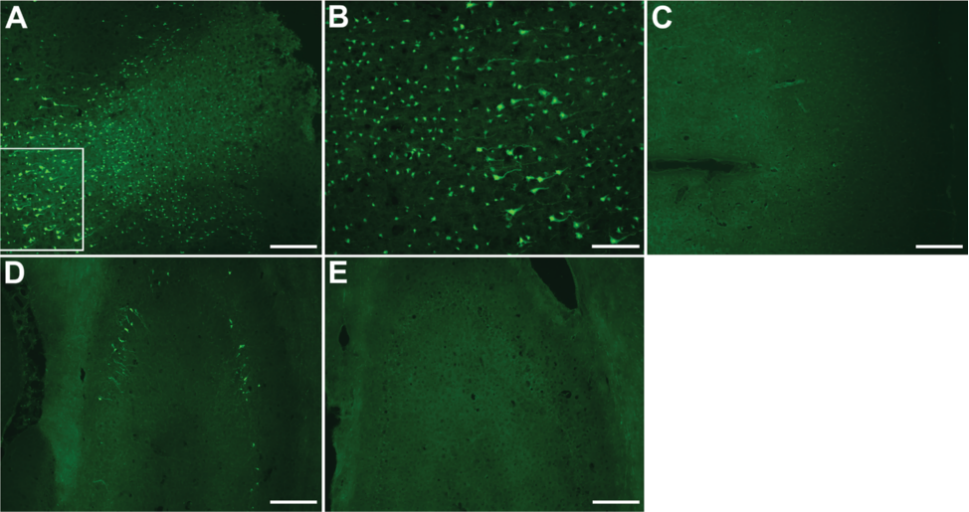
Fluoro-Jade® B staining of the cortex and hippocampus. Fluoro-Jade® B (FJB) staining showed a dense distribution of damaged neurons throughout all layers of the cortex near the sight of impact (**a**, **b**). Damaged neurons were also detected in the hippocampus ipsilateral to the injury (**d**). These neurons were sparsely distributed in the hippocampal CA regions. No FJB staining was detected in either brain region contralateral to the injury (C: cortex; E: hippocampus). *FJB: green; Scale bars: 200 μm (* ***a*** *,* ***c*** *-* ***e*** *), 100 μm (* ***b*** *)*

To examine cell death histologically, we chose to look at the cortical area adjacent to the impact site so we could observe the cellular response to the injury in all layers of the cortex. This is not possible at the impact site because of the resulting injury cavity. Fluoro-Jade® B (FJB) staining showed a dense distribution of damaged neurons throughout all layers of the cortex near the sight of impact (Fig. 2a, b). Damaged neurons were also detected in the hippocampus ipsilateral to the injury (Fig. 2d). These neurons were sparsely distributed in the hippocampal CA regions. No FJB staining was detected in the cortex (Fig. 2c) or hippocampus (Fig. 2e) contralateral to the injury.

**Fig. 3:**
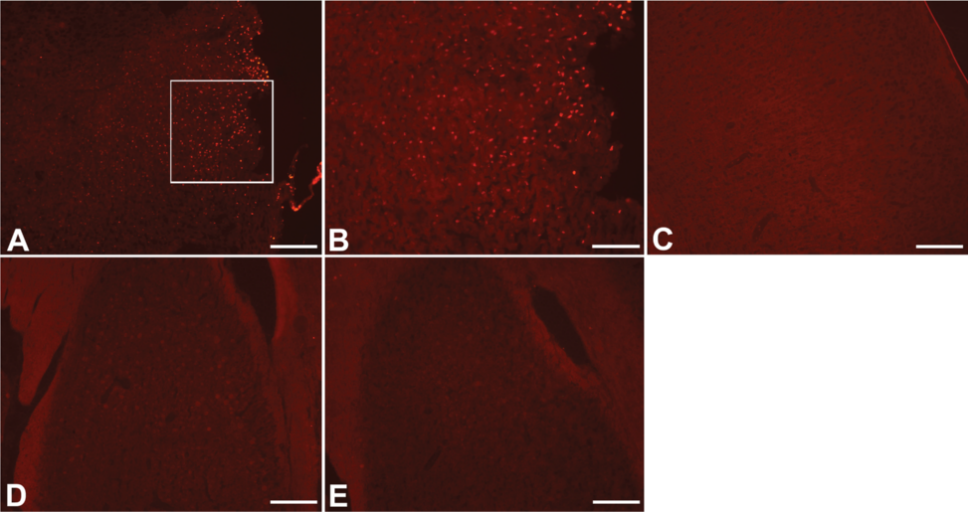
TUNEL staining of the cortex and hippocampus. TUNEL staining showed distribution of injured cells in the cortex similar to FJB as they were distributed throughout all layers of the cortex (**a**, **b**). However, no TUNEL staining was detected in the ipsilateral hippocampus (**d**). No TUNEL was observed on the contralateral side of the brain (C: cortex; E: hippocampus). *TUNEL: red; Scale bars: 200 μm (* ***a*** *,* ***c*** *-* ***e*** *), 100 μm (* ***b*** *)*

TUNEL staining showed distribution of injured cells in the cortex similar to FJB as they were distributed throughout all layers of the cortex (Fig. 3a, b). However, no TUNEL staining was detected in the ipsilateral hippocampus (Fig. 3d), suggesting that the neuronal damage in that region had not yet progressed to apoptosis. No TUNEL was observed in the contralateral cortex (Fig. 3c) or hippocampus (Fig. 3e).

### Cell death gene expression patterns

**Fig. 4:**
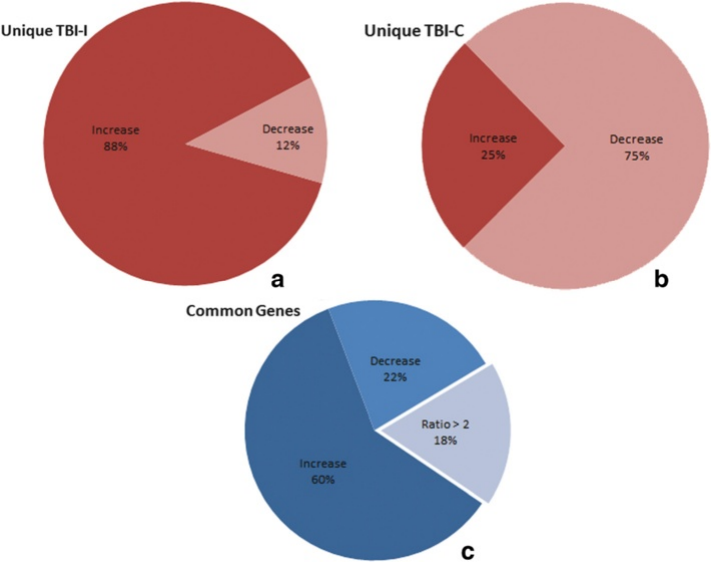
Breakdown of CD genes based on increased and decreased expression. **a** 361 CD genes changed uniquely on the ipsilateral side of the brain and 88 % (317 genes) of those increased in expression. **b** 136 CD genes changed uniquely on the contralateral side of the brain and 75 % (102 genes) of those decreased in expression. **c** There were 405 genes that changed more than 2-fold on both sides of the brain. Eighty-two percent of them (332 genes) changed similarly while the remaining 18 % (73 genes) changed differently (TBI-I/TBI-C ratio >2; see text)

Focusing on the CD genes in our datasets, we determined that 902 CD genes had a greater than 2-fold change in expression. Of these genes, 361 CD genes changed uniquely on the ipsilateral side of the brain. 317 of those genes (88 %) increased while 44 genes (12 %) decreased in expression (Fig. 4a). 136 CD genes changed uniquely on the contralateral side of the brain and, in contrast to what we observed on the ipsilateral side, only 34 genes (25 %) increased while 102 genes (75 %) decreased in expression (Fig. 4b).

There were 405 CD genes that changed on both the ipsilateral and contralateral sides of the brain. In order to determine whether these common genes changed differently on one side of the brain compared to the other, we calculated the ratio of the TBI-I fold change to the TBI-C fold change. Those genes that had a TBI-I/TBI-C ratio greater than 2 were determined to have changed differently. We observed that 332 of the common CD genes (82 %) changed similarly (TBI-I/TBI-C ratio < 2; Fig. 4c). Of the genes that changed similarly, 242 genes (60 %) increased in expression and 90 genes (22 %) decreased in expression. The remaining 73 common CD genes (18 %) changed differently (TBI-I/TBI-C ratio > 2) (Fig. 4c). Table 1 shows the 73 common CD genes that changed differently. These genes span all cellular compartments (extracellular space, plasma membrane, cytoplasm, and nucleus) with diverse molecule types. The expression of all these genes was lower on the contralateral side of the brain with the exception of 3 genes, DNAJB6, TRIM54 and PSIP1 (negative TBI-I/TBI-C ratio). Because of their different expression patterns, these 73 genes became our first group of genes of interest (GOI; Table 1). Notable genes given their high TBI-I/TBI-C ratio included SPP1, TIMP1, LCN2, SERPINA3, KCNN4, HSPB1, RDX, Slpi, ATRX, DNAJB6, NAA15, SMARCA4, STAT3, and THOC2.

**Table 1:** Genes that change differently on each side of the brain

Gene symbol	Entrez gene name	TBI-I fold change	TBI-C fold change	TBI-I/TBI-C ratio	Molecular type
*Extracellular Space*					
SPP1	secreted phosphoprotein 1	37.905	2.370	15.994	cytokine
TIMP1	TIMP metallopeptidase inhibitor 1	38.486	2.101	18.318	cytokine
CP	ceruloplasmin (ferroxidase)	27.838	8.477	3.284	enzyme
FGL2	fibrinogen-like 2	16.793	4.017	4.180	peptidase
LCN2	lipocalin 2	71.824	3.895	18.440	transporter
SERPINA3	serpin peptidase inhibitor, clade A (alpha-1 antiproteinase, antitrypsin), member 3	58.488	2.509	23.311	other
*Plasma Membrane*					
CD44	CD44 molecule (Indian blood group)	15.558	2.399	6.485	enzyme
EHD4	EH-domain containing 4	2.361	−2.056	4.854	enzyme
SDC1	syndecan 1	13.681	2.566	5.332	enzyme
KCND2	potassium voltage-gated channel, Shal-related subfamily, member 2	−2.792	−7.585	2.717	ion channel
KCNN4	potassium intermediate/small conductance calcium-activated channel, subfamily N, member 4	3.088	−9.429	29.117	ion channel
CAMK2N1	calcium/calmodulin-dependent protein kinase II inhibitor 1	−11.813	−23.824	2.017	kinase
EGFR	epidermal growth factor receptor	6.773	2.374	2.853	kinase
PTPRF	protein tyrosine phosphatase, receptor type, F	−6.365	−20.492	3.219	phosphatase
IL6ST	interleukin 6 signal transducer	2.307	−3.283	7.574	transmembrane receptor
CD68	CD68 molecule	4.365	2.007	2.175	other
HLA-A	major histocompatibility complex, class I, A	9.296	3.657	2.542	other
PMEPA1	prostate transmembrane protein, androgen induced 1	2.682	−2.937	7.877	other
*Cytoplasm*					
CYP1B1	cytochrome P450, family 1, subfamily B, polypeptide 1	10.998	4.808	2.287	enzyme
KIF3A	kinesin family member 3A	−5.083	−11.754	2.312	enzyme
MX1	MX dynamin-like GTPase 1	28.177	7.326	3.846	enzyme
PDE4B	phosphodiesterase 4B, cAMP-specific	5.602	2.359	2.375	enzyme
RND3	Rho family GTPase 3	2.864	−2.971	8.509	enzyme
SRXN1	sulfiredoxin 1	6.306	2.402	2.625	enzyme
CARD11	caspase recruitment domain family, member 11	7.343	2.892	2.539	kinase
CSNK2A1	casein kinase 2, alpha 1 polypeptide	2.992	−2.750	8.228	kinase
EIF5B	eukaryotic translation initiation factor 5B	−3.044	−8.766	2.880	translation regulator
RASA1	RAS p21 protein activator (GTPase activating protein) 1	2.392	−2.105	5.035	transporter
AHI1	Abelson helper integration site 1	2.243	−2.897	6.498	other
CISD2	CDGSH iron sulfur domain 2	−7.833	−19.012	2.427	other
CMIP	c-Maf inducing protein	−3.778	−13.763	3.643	other
Ctdspl	CTD (carboxy-terminal domain, RNA polymerase II, polypeptide A) small phosphatase-like	−7.271	−36.886	5.073	other
HSPB1	heat shock 27 kDa protein 1	46.922	2.639	17.780	other
KIFAP3	kinesin-associated protein 3	−2.281	−7.831	3.433	other
LCP1	lymphocyte cytosolic protein 1 (L-plastin)	6.082	2.799	2.173	other
LSP1	lymphocyte-specific protein 1	11.716	2.140	5.475	other
PHLDA1	pleckstrin homology-like domain, family A, member 1	5.129	2.160	2.375	other
RDX	radixin	4.828	−5.274	25.463	other
Slpi	secretory leukocyte peptidase inhibitor	82.908	3.119	26.582	other
Tpm3	tropomyosin 3	2.592	−2.715	7.037	other
TRIM54	tripartite motif containing 54	−4.426	−2.032	−2.178	other
*Nucleus*					
SETD8	SET domain containing (lysine methyltransferase) 8	2.029	−3.930	7.974	enzyme
TOP2A	topoisomerase (DNA) II alpha 170 kDa	2.260	−2.406	5.438	enzyme
CDK11A	cyclin-dependent kinase 11A	−4.290	−14.872	3.467	kinase
GSK3B	glycogen synthase kinase 3 beta	−2.733	−6.635	2.428	kinase
SRPK2	SRSF protein kinase 2	−5.614	−23.589	4.202	kinase
THRA	thyroid hormone receptor, alpha	−2.799	−11.518	4.115	ligand-dependent nuclear receptor
ATRX	alpha thalassemia/mental retardation syndrome X-linked	2.091	−5.964	12.471	transcription regulator
BTG2	BTG family, member 2	−2.220	−5.803	2.614	transcription regulator
CCAR1	cell division cycle and apoptosis regulator 1	−2.943	−11.648	3.958	transcription regulator
CCND1	cyclin D1	2.152	−2.027	4.362	transcription regulator
CEBPD	CCAAT/enhancer binding protein (C/EBP), delta	11.271	2.037	5.533	transcription regulator
DEK	DEK proto-oncogene	−3.006	−7.352	2.446	transcription regulator
DNAJB6	DnaJ (Hsp40) homolog, subfamily B, member 6	−4.383	5.614	−24.606	transcription regulator
KLF13	Kruppel-like factor 13	−2.006	−4.582	2.284	transcription regulator
KLF6	Kruppel-like factor 6	6.003	2.865	2.095	transcription regulator
NAA15	N(alpha)-acetyltransferase 15, NatA auxiliary subunit	3.605	−3.751	13.522	transcription regulator
NFIX	nuclear factor I/X (CCAAT-binding transcription factor)	−2.548	−8.112	3.184	transcription regulator
PA2G4	proliferation-associated 2G4, 38 kDa	−2.702	−5.783	2.140	transcription regulator
SMARCA4	SWI/SNF related, matrix associated, actin dependent regulator of chromatin, subfamily a, member 4	2.521	−7.712	19.442	transcription regulator
STAT3	signal transducer and activator of transcription 3 (acute-phase response factor)	4.219	−3.771	15.910	transcription regulator
TBL1XR1	transducin (beta)-like 1 X-linked receptor 1	2.587	−2.134	5.521	transcription regulator
TCF4	transcription factor 4	−2.216	−4.625	2.087	transcription regulator
TPR	translocated promoter region, nuclear basket protein	2.212	−2.728	6.034	transporter
Brd4	bromodomain containing 4	−3.528	−15.202	4.309	other
CDT1	chromatin licensing and DNA replication factor 1	3.098	−2.295	7.110	other
GADD45G	growth arrest and DNA-damage-inducible, gamma	3.191	−2.384	7.607	other
PSIP1	PC4 and SFRS1 interacting protein 1	−2.663	2.113	−5.627	other
Rbm25	RNA binding motif protein 25	−5.547	−16.213	2.923	other
THOC2	THO complex 2	2.119	−4.886	10.353	other
*Unknown*					
EIF3C	eukaryotic translation initiation factor 3, subunit C	−4.369	−9.072	2.076	translation regulator
Nos1ap	nitric oxide synthase 1 (neuronal) adaptor protein	−2.698	−5.717	2.119	other
RASSF4	Ras association (RalGDS/AF-6) domain family member 4	4.289	2.106	2.037	other

TBI-I/TBI-C Ratio: Gene increased on both sides (TBI-I > TBI-C): ratio = (TBI-I)/(TBI-C); Gene decreased on both sides (TBI-I > TBI-C): ratio = 1/[(TBI-I)/(TBI-C)]; Gene decreased on both sides (TBI-I < TBI-C): ratio = −1/[(TBI-C)/(TBI-I)]; Gene increased ipsilaterally and decreased contralaterally: ratio = (TBI-I)/-[1/(TBI-C)]; Gene decreased ipsilaterally and increased contralaterally: ratio = (TBI-C)/[1/(TBI-I)]

### Canonical pathway analysis

**Fig. 5:**
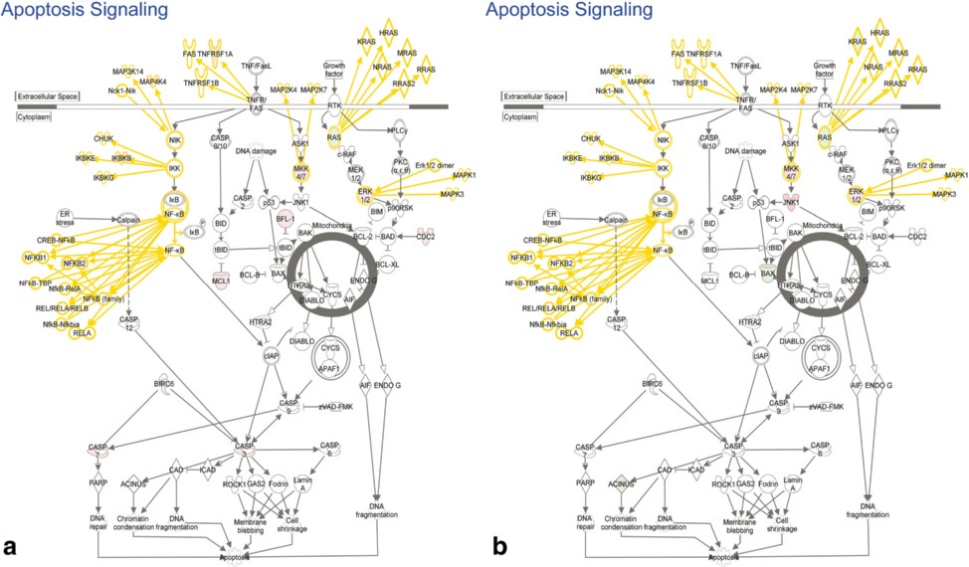
Canonical pathway analysis. The apoptosis signaling pathway with all gene families, groups and complexes expanded to show the member genes and showing the relative expression values of potential GOI for TBI-I (**a**) and TBI-C (**b**) included in this pathway. *red: relative increase in expression; green: relative decrease in expression; white: no change in expression; gold connections and outlines: expansion of gene families, groups and complexes in the original pathway*

We used canonical pathway and network analysis in IPA to identify genes in our datasets that were potentially most relevant to the observed CD gene response. We defined potential GOI, in this context, as those genes that either changed in expression uniquely on one side of the brain, or were one of the 73 common genes that changed differently (Table 1). GOI were identified by comparing the genes in the canonical pathway and gene networks to the list of unique TBI-I or TBI-C CD genes with the genes from Table 1 added to each list and identifying the overlapping genes. Canonical pathways in IPA are well-characterized metabolic and cell signaling pathways derived from information found in specific journal articles, review articles, text books, and KEGG Ligand [21]. Fig. 5 shows the apoptosis signaling canonical pathway with all relevant gene families, groups and complexes expanded to show the member genes. This pathway was chosen because apoptosis is a key process in cell death following TBI [22–24]. By overlaying the relative expression values of potential GOI for TBI-I (Fig. 5a) and TBI-C (Fig. 5b), we were able to identify 9 GOI that were increased (BCL2A1 (Bfl-1 in pathway), CASP3, CASP7, CDK1 (Cdc2), IKBKB, MAP4K4, MCL1, NFKB2, and TNFRSF1A) in the TBI-I dataset, 3 GOI that decreased (ACIN1 (Acinus), BAX and KRAS) and 1 GOI that increased (MAPK8 (JNK1)) in the TBI-C dataset.

### Gene network analysis

In contrast to canonical pathways, which are relatively immutable in IPA, gene networks are generated *de novo* in IPA based on the list of genes that are imported. IPA takes “seed” molecules from the gene list, searches the Ingenuity Knowledge Base, and uses a network algorithm to draw connections between molecules based on biological function [25]. In order to generate the networks, we performed an IPA core analysis on the TBI-I and TBI-C CD datasets. IPA scores the networks in order to rank them according to their degree of relevance to the network eligible molecules in the dataset [25]. The top 6 scoring networks for each dataset were used to identify GOI.

**Fig. 6:**
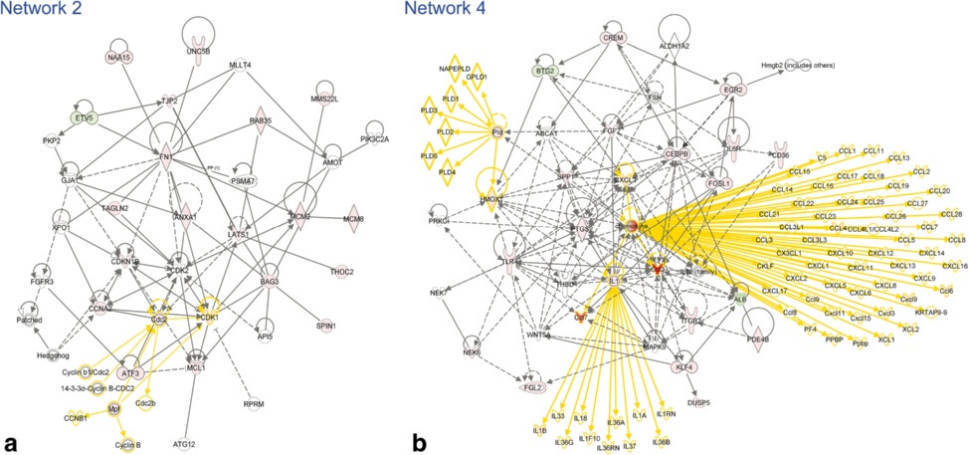
Examples of TBI-I networks. TBI-I CD networks 2 (**a**) and 4 (**b**) (see Table 2) with all gene families, groups and complexes expanded to show the member genes and showing the relative expression values of potential GOI for TBI-I. *red: relative increase in expression; green: relative decrease in expression; white: no change in expression; gold connections and outlines: expansion of gene families, groups and complexes in the original network*

**Fig. 7:**
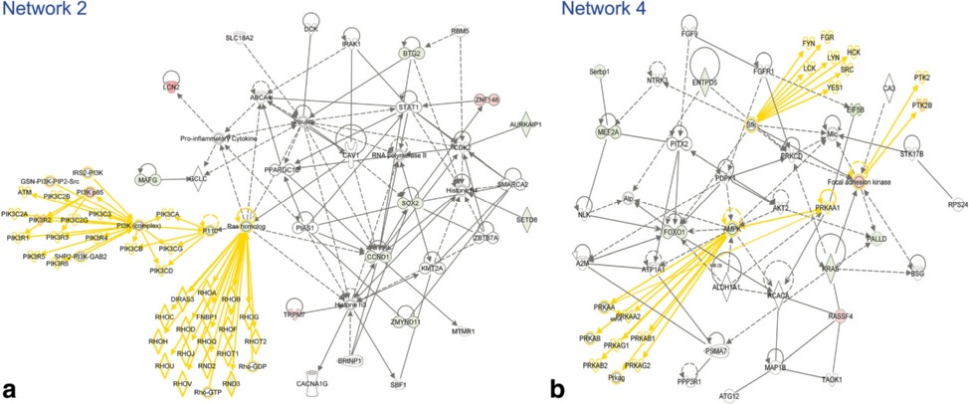
Examples of TBI-C networks. TBI-C CD networks 2 (**a**) and 4 (**b**) (see Table 3) with all gene families, groups and complexes expanded to show the member genes and showing the relative expression values of potential GOI for TBI-C. *red: relative increase in expression; green: relative decrease in expression; white: no change in expression; gold connections and outlines: expansion of gene families, groups and complexes in the original network*

Five of the top 6 networks for TBI-I and all 6 networks for TBI-C have cell death and survival as their top associated biological function (Tables 2 and 3). Only TBI-I network 4 does not have cell death and survival as one of the top 3 associated biological functions. Figure 6 shows networks 2 and 4 (Table 2) as examples of the TBI-I analysis. Figure 7 shows networks 2 and 4 (Table 3) as examples of the TBI-C analysis. (The other networks are available as supplemental materials (Additional files 1 and 2).) Like the canonical pathway analysis, all relevant gene families, groups and complexes were expanded to show the member genes. The relative TBI-I (Fig. 6) and TBI-C (Fig. 7) gene expression values of potential GOI were overlaid on these networks and additional GOI were identified. Tables 4 and 5 show the resulting GOI that were identified through this analysis. For TBI-I, a total of 110 GOI were found in these networks, 22 of which were previously identified (Table 4). Thus, 88 additional GOI were identified for TBI-I. For TBI-C, 38 additional GOI were identified as 28 of the 66 GOI found had been previously identified (Table 5). The most prevalent molecular types for TBI-I were transcription regulators, unspecified enzymes, kinases, and undefined molecules. Kinases transcription regulators, unspecified enzymes, and undefined molecules were most prevalent in the TBI-C analysis.

**Table 2:** The top 6 gene networks associated with the TBI-I dataset

Network ID	Molecules in network	Score	Focus molecules	Top diseases and functions
1	**CADM1, CALB1, CBFB, CDCA7L, CMIP,** Cytochrome bc1**,** cytochrome-c oxidase**, DAB2, DEDD, FGF9, FLNA, FYN, GCLC, GCLM, GFAP, GFRA1, ITGA6, JDP2, MAOA, MED14, MGEA5, NFE2L1, NFE2L2, NPTX1, NRP1, PDHA1, PDLIM7, RET,** Rnr**, RPS24, RTN4, SLC18A2,** Sos**, STK17B, TAF4B**	46	31	Cell Death and Survival, Drug Metabolism, Molecular Transport
2	**AMOT, ANXA1, API5, ATF3, ATG12, BAG3, CCNA2,** Cdc2**, CDK1, CDK2, CDKN1B, ETV5, FGFR3, FN1, GJA1,** Hedgehog**, LATS1, MCL1, MCM2, MCM8, MLLT4, MMS22L, NAA15,** Patched**, PIK3C2A, PKP2, PSMA7, RAB35, RPRM, SPIN1, TAGLN2, THOC2, TJP2, UNC5B, XPO1**	46	32	Cell Death and Survival, Cell Cycle, Reproductive System Development and Function
3	**AHCTF1, AKAP12,** amylase**, BCL11A, CA4, CACNA1G, CCND1, CLCN7, CREB1, CREBBP, CSF1, CSRNP1, CTNNB1, DES,** Histone h3**,** IKK (complex)**, ITPR2, KLF6, KPNB1, MITF, MTMR1, NFIX, PRKD3, PTGR1, RAI14,** RNA polymerase II**, RRM2, SENP2, SMAD4, SMARCA4, SUDS3, TBL1XR1, TGM2, THRA, ZBTB18**	43	31	Cell Death and Survival, Organismal Survival, Gene Expression
4	**ABCA1, ALB, ALDH1A2, BTG2, Ccl2, Ccl7, CD36, CEBPB,** chemokine**, CREM, CXCL3, DUSP5, EGR2, FGF2, FGL2, FOSL1,** FSH**, Hmgb2 (includes others), HMOX1,** IL1**,** IL12 (family)**, IL6R, ITGB2, KLF4, MAPK9, NEK6, NEK7, PDE4B,** Pld**, PRKCI, PTGS2, SPP1, THBD, TLR4, WNT5A**	42	30	Cellular Movement, Hematological System Development and Function, Immune Cell Trafficking
5	**ACSL5, AGTR2, AMFR, AVP, CAMK2N1, CAPRIN1, CHSY1, CUL5, DCK, ELAVL1,** Endothelin**, GMCL1,** GNRH**,** Insulin**, MAP4K4, MSI2,** MTORC1**, NEO1, OPA1,** Proinsulin**, PTGER3,** Relaxin**, RNF2, SLC2A3, SMAD7, STAG1, TACR1, TCEB3, TMEM123, TRAF6, WAPAL, WFS1, WTAP, ZMYM2, ZNF280B**	41	29	Cell Death and Survival, Cardiovascular System Development and Function, Hereditary Disorder
6	26 s Proteasome**, ARL11, BCL2L1, CAMK1G, CAMK2D, CASP3, CAV1, CISD2, CLASP1, CLN5, DLG4, EN2, ENC1,** Esr1-Esr1-estrogen-estrogen**, FBXO9, G2E3,** Hsp70**,** Hsp90**, HSP90AB1, IDE, KIF1B, MDM2, PCDH15, PGR, PI4K2A, PRDM2, PSEN1, SGPL1, SNCA, SPTBN1,** SRC (family)**, SRPK2, TMEM109, TRIM2, VPS41**	40	30	Cell Death and Survival, Cancer, Neurological Disease

Bold= > Gene included in the dataset

Note: Some of the nodes in the original networks represent gene groups, complexes or families that, when expanded, contain more potential GOI

**Table 3:** The top 6 gene networks associated with the TBI-C dataset

Network ID	Molecules in network	Score	Focus molecules	Top diseases and functions
1	**ACER2, ACIN1, ACVR1C, ALDH1A2, ARHGEF7, BCL11B,** caspase**, CBFB, CD38, CD44, CLCN3, CUL5, DPYD, EEF1A2, FGL2,** Fibrinogen**, ITGB1, MAP3K1, MAP3K8, MAPK8, MAPK9, MIF, MTDH, PAK1, PRDX6,** Rac**, RAD23B, SPARC,** Srebp**, TNKS2, TTLL1, VCL, WNT5A, ZBTB18, ZYX**	50	31	Cell Death and Survival, Cellular Movement, Ophthalmic Disease
2	**ABCA1, AURKAIP1, BRINP1, BTG2, CACNA1G, CAV1, CCND1, CDK2, DCK, GCLC,** Histone h3**,** Histone h4**,** Insulin**, IRAK1, KMT2A, LCN2, MAFG, MTMR1,** P110**, PIAS1, PPARGC1B,** Pro-inflammatory Cytokine**,** Ras homolog**, RBM5,** RNA polymerase II**, SBF1, SETD8, SLC18A2, SMARCA2, SOX2, STAT1, TRPM7, ZBTB7A, ZMYND11, ZNF148**	44	28	Cell Death and Survival, Gene Expression, Cellular Growth and Proliferation
3	**ADNP, AHI1, ANKS1B, ARL6IP1, CDK11A, CXCL12, DNAJB6, ENC1,** estrogen receptor**, FBXO9, FBXW7, FGFR3, G2E3,** Hdac**,** HSP**,** Hsp90**, HSP90AA1, HSP90AB1, HSPB1, KLF9, KLF13, LINGO1, MED1, MED14,** mediator**, PA2G4, PGR, PPP3CB, RNF4, STUB1, THRA,** TRAP/Media**, TUFM,** Ubiquitin**, VPS41**	43	28	Cell Death and Survival, Post-Translational Modification, Protein Folding
4	**A2M, ACACA, AKT2, ALDH1A1,** Alp**,** AMPK**, ATG12, ATP1A1, BSG, CA3, EIF5B, ENTPD5, FGF9, FGFR1,** Focal adhesion kinase**, FOXO1, KRAS, MAP1B, MEF2A,** Mlc**, NLK, NTRK3, PALLD, PDPK1, PITX2, PPP3R1, PRKAA1, PRKCD, PSMA7, RASSF4, RPS24, Serbp1,** Sfk**, STK17B, TAOK1**	41	30	Cell Death and Survival, Carbohydrate Metabolism, Cellular Development
5	ACAC**, AP2B1, APAF1,** APC-AXIN-GSK3β**, ATP2A2, ATP2B1, ATP2B2, BAX,** Ca2 ATPase**,** calpain**, CAST, CDH13,** Cytochrome bc1**,** cytochrome C**,** cytochrome-c oxidase**, DDIT4, DNM1L, GBX2,** glutathione peroxidase**, GSK3B, ITSN1, KCND2, LMO4, MAFB, MAOA, MFN1,** Mitochondrial complex 1**, MTF2, NCS1, NDUFAB1, NFE2L1, OPA1, PACS2, PEX11B, PRKAA2**	39	26	Cell Death and Survival, Cell Cycle, Cellular Compromise
6	Ap1**, ARHGAP1, ARL6IP5, CCDC86, CCND2, CEBPD,** Cg**, COL1A1, DACH1,** FSH**,** Growth hormone**,** Gsk3**, IGFBP3,** Lh**, MGEA5, NEO1, PDHA1, PPP2R1A, PRLR, PSIP1, PURA, RAB27A, RPRM, RSF1, SMAD4, SMAD7,** Smad1/5/8**,** Smad2/3**, SP1, SPP1, TAF4B,** Tgf beta**, TIMP1, TNRC6A, ZMYM2**	39	26	Cell Death and Survival, Tissue Development, Cellular Growth and Proliferation

Bold= > Gene included in the dataset

Note: Some of the nodes in the original networks represent gene groups, complexes or families that, when expanded, contain more potential GOI

**Table 4:** Identification of genes of interest from TBI-I network analysis

Network ID	GOI found	Total # of GOI	Overlap with previous analyses	Net # of GOI	Top molecular types
1	**CALB1, CDCA7L,** *CMIP* **, DAB2, FLNA, GCLM, GFAP, NFE2L2, PDLIM7**	9	1	8	undefined
2	**ANXA1, ATF3, BAG3, CCNA2,** *CDK1* **, CDKN1B, ETV5, FN1, LATS1,** *MCL1* **, MCM2, MCM8, MMS22L,** *NAA15* **, RAB35, SPIN1, TAGLN2,** *THOC2* **, TJP2, UNC5B**	20	4	16	undefined, enzymes, and kinases
3	**BCL11A,** *CCND1* **, CREB1, CREBBP, CSRNP1, DES,** *IKBKB* **, ITPR2,** *KLF6* **, KPNB1, MITF,** *NFIX* **, PTGR1, RAI14, RRM2, SENP2,** *SMARCA4* **, SUDS3,** *TBL1XR1* **, TGM2,** *THRA*	21	7	14	transcription regulators and enzymes
4	**ALB,** *BTG2* **, Ccl2, CCL3L3, CCL4, Ccl6, Ccl7, CD36, CEBPB, CREM, CX3CL1, CXCL3, Cxcl9, DUSP5, EGR2, FGF2,** *FGL2* **, FOSL1, HMOX1, IL1B, IL6R, ITGB2, KLF4, NEK6,** *PDE4B* **, PTGS2,** *SPP1* **, TLR4**	28	4	24	cytokines, transcription regulators, and transmembrane receptors
5	**ACSL5,** *CAMK2N1* **, CHSY1, ELAVL1,** *MAP4K4* **, MSI2, PTGER3, TCEB3, TMEM123, TRAF6, WFS1**	11	2	9	undefined and kinases
6	**ARL11, CAMK1G,** *CASP3* **,** *CISD2* **, CLN5, DNAJB6,** *DNAJB9* **, FGR, HCK, HSPA1A/HSPA1B, HSPA2, HSPA9, HSPB8, MDM2, PCDH15, PI4K2A, PRDM2, SGPL1, SNCA,** *SRPK2* **, TMEM109**	21	4	17	undefined, kinases, and transcription regulators

Italics= > gene of interest also found in a previous analysis; Bold= > GOI unique to this analysis

**Table 5:** Identification of genes of interest from TBI-C network analysis

Network ID	GOI found	Total # of GOI	Overlap with previous analyses	Net # of GOI	Top molecular types
1	*ACIN1* **, ACVR1C,** *CD44* **, DPYD,** *FGL2* **,** *MAPK8* **, MTDH, RAD23B, TTLL1**	9	4	5	enzymes and kinases
2	**AURKAIP1,** *BTG2* **,** *CCND1* **,** *LCN2* **, MAFG, PIK3CD, PIK3R2,** *RND3* **,** *SETD8* **, SOX2, TRPM7, ZMYND11, ZNF148**	13	5	8	transcription regulators, kinases, and enzymes
3	*AHI1* **,** *CDK11A* **, CDK19,** *DNAJB6* **, HSP90AA1,** *HSPB1* **,** *KLF13* **, LINGO1, MED1,** *PA2G4* **, PPP3CB,** *THRA* **, TUFM**	13	7	6	transcription regulators, undefined, and kinases
4	*EIF5B* **, ENTPD5, FOXO1,** *KRAS* **, MEF2A, PALLD, PRKAA2, PTK2B,** *RASSF4* **, Serbp**	10	3	7	undefined, transcription regulators, enzymes, and kinases
5	**ATP2A2, ATP2B2,** *BAX* **, CDH13, GBX2,** *GSK3B* **,** *KCND2* **, MAFB, MFN1, NDUFAB1,** *PRKAA2*	11	4	7	transporters, kinases, enzymes, and undefined
6	**CCDC86, CCND2,** *CEBPD* **,** *GSK3B* **, PRLR,** *PSIP1* **, RSF1, SP1,** *SPP1* **,** *TIMP1*	10	5	5	transcription regulators, undefined, and cytokines

Italics= > gene of interest also found in a previous analysis; Bold= > GOI unique to this analysis

### Compiling the gene interaction hierarchy (GIH)

**Fig. 8:**
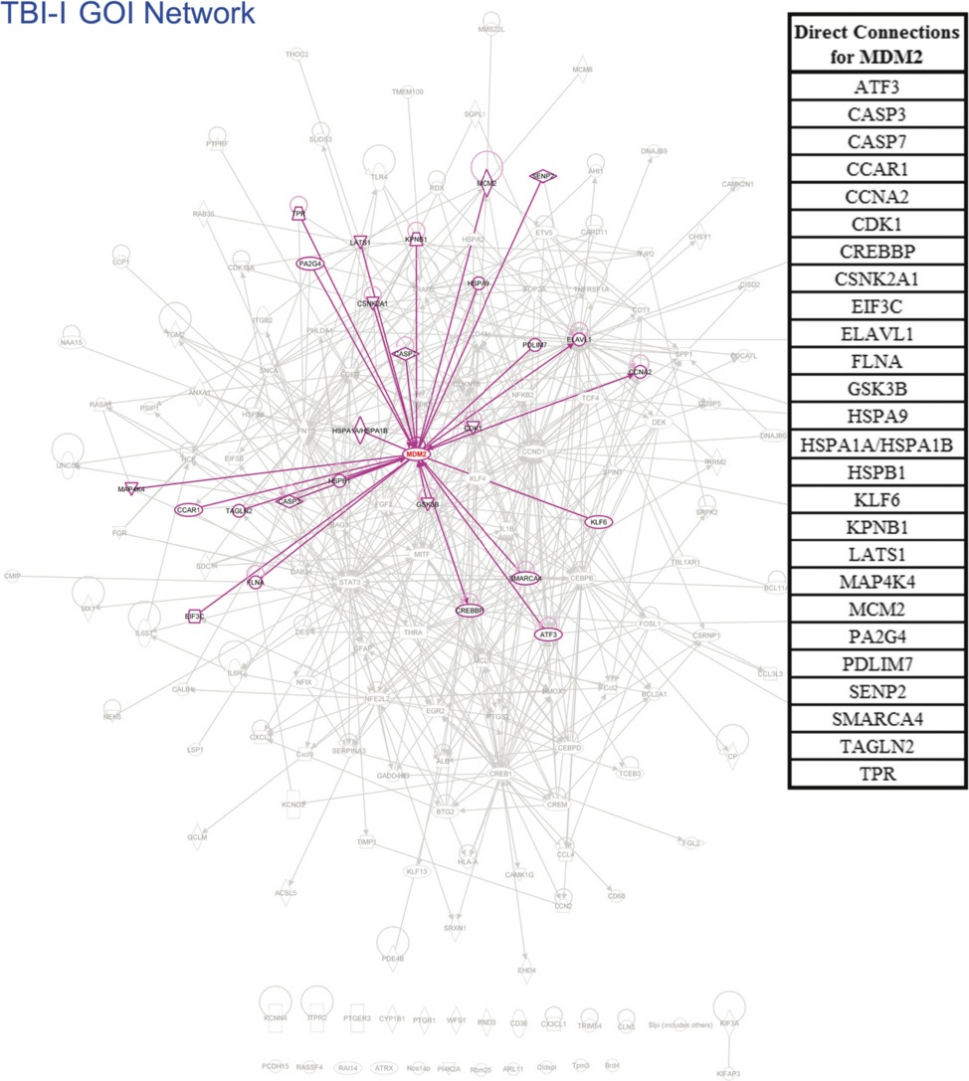
An example of calculating the number of direct connections for the TBI-I GOI network. In IPA, the gene in question was selected (MDM2 in this example). Then, its direct connections were selected by right clicking on MDM2 and using the “select nearest neighbors” option (highlighted in purple). A list of the selected genes was exported and MDM2 was removed from the list (upper right corner). The remaining genes were counted (26 in this example) and MDM2 was ranked in the TBI-I gene interaction hierarchy (primary tier) by this number

TBI-I: By combining the GOI identified through canonical pathway and network analysis with those in Table 1, we identified a total of 170 GOI. In order to determine which genes might be most relevant to CD, we ranked these genes relative to each other by the number of direct interactions each had with the other GOI. Our analysis showed that 145 of the GOI formed an interconnected network, leaving 25 “orphan” genes (see Additional file 3). Genes having 1st order connections with more than 10 % of the other genes within the main GOI network (>14 connections) were considered “primary” in this analysis (see Fig. 8 for an example). Genes having connections with 5 %–10 % of the other genes (8–14 connections) were considered “secondary” (see Additional file 4 for an example) and those with connections with less than 5 % of the other genes (<8 connections) were considered “peripheral”. The resultant GIH is displayed in Table 6.

**Table 6:** TBI-I Gene interaction hierarchy (GIH)

Gene symbol	Entrez gene name	Fold change	Cellular compartment	Molecular type
*Primary*				
ATF3	activating transcription factor 3	12.027	Nucleus	transcription regulator
*CCND1*	cyclin D1	2.152	Nucleus	transcription regulator
CEBPB	CCAAT/enhancer binding protein (C/EBP), beta	3.366	Nucleus	transcription regulator
CREB1	cAMP responsive element binding protein 1	2.666	Nucleus	transcription regulator
CREBBP	CREB binding protein	2.421	Nucleus	transcription regulator
MDM2	MDM2 proto-oncogene, E3 ubiquitin protein ligase	2.01	Nucleus	transcription regulator
NFE2L2	nuclear factor, erythroid 2-like 2	2.452	Nucleus	transcription regulator
*SMARCA4*	SWI/SNF related, matrix associated, actin dependent regulator of chromatin, subfamily a, member 4	2.521	Nucleus	transcription regulator
*STAT3*	signal transducer and activator of transcription 3 (acute-phase response factor)	4.219	Nucleus	transcription regulator
CDK1	cyclin-dependent kinase 1	2.105	Nucleus	kinase
*CSNK2A1*	casein kinase 2, alpha 1 polypeptide	2.992	Cytoplasm	kinase
*EGFR*	epidermal growth factor receptor	6.773	Plasma Membrane	kinase
*GSK3B*	glycogen synthase kinase 3 beta	−2.733	Nucleus	kinase
*CD44*	CD44 molecule (Indian blood group)	15.558	Plasma Membrane	enzyme
FN1	fibronectin 1	3.97	Extracellular Space	enzyme
TRAF6	TNF receptor-associated factor 6, E3 ubiquitin protein ligase	2.163	Cytoplasm	enzyme
CASP3	caspase 3, apoptosis-related cysteine peptidase	2.535	Cytoplasm	peptidase
ELAVL1	ELAV like RNA binding protein 1	3.275	Cytoplasm	other
*Secondary*				
*CEBPD*	CCAAT/enhancer binding protein (C/EBP), delta	11.271	Nucleus	transcription regulator
CREM	cAMP responsive element modulator	2.165	Nucleus	transcription regulator
EGR2	early growth response 2	2.271	Nucleus	transcription regulator
FOSL1	FOS-like antigen 1	5.875	Nucleus	transcription regulator
KLF4	Kruppel-like factor 4 (gut)	2.057	Nucleus	transcription regulator
MITF	microphthalmia-associated transcription factor	4.755	Nucleus	transcription regulator
*TCF4*	transcription factor 4	−2.216	Nucleus	transcription regulator
HSPA1A/HSPA1B	heat shock 70 kDa protein 1A	3.137	Cytoplasm	enzyme
MCM2	minichromosome maintenance complex component 2	2.57	Nucleus	enzyme
PTGS2	prostaglandin-endoperoxide synthase 2 (prostaglandin G/H synthase and cyclooxygenase)	3.106	Cytoplasm	enzyme
IL1B	interleukin 1, beta	5.166	Extracellular Space	cytokine
*SPP1*	secreted phosphoprotein 1	37.905	Extracellular Space	cytokine
CDKN1B	cyclin-dependent kinase inhibitor 1B (p27, Kip1)	3.732	Nucleus	kinase
IKBKB	inhibitor of kappa light polypeptide gene enhancer in B-cells, kinase beta	2.127	Cytoplasm	kinase
KPNB1	karyopherin (importin) beta 1	3.173	Nucleus	transporter
MCL1	myeloid cell leukemia 1	3.25	Cytoplasm	transporter
*THRA*	thyroid hormone receptor, alpha	−2.799	Nucleus	ligand-dependent nuclear receptor
CASP7	caspase 7, apoptosis-related cysteine peptidase	2.579	Cytoplasm	peptidase
BAG3	BCL2-associated athanogene 3	4.045	Cytoplasm	other
CCNA2	cyclin A2	2.633	Nucleus	other
GFAP	glial fibrillary acidic protein	3.011	Cytoplasm	other
HSPA9	heat shock 70 kDa protein 9 (mortalin)	2.666	Cytoplasm	other
*HSPB1*	heat shock 27 kDa protein 1	46.922	Cytoplasm	other
SNCA	synuclein, alpha (non A4 component of amyloid precursor)	−2.169	Cytoplasm	other
*Peripheral*				
ACSL5	acyl-CoA synthetase long-chain family member 5	−2.361	Cytoplasm	enzyme
ANXA1	annexin A1	3.535	Plasma Membrane	enzyme
CHSY1	chondroitin sulfate synthase 1	2.873	Cytoplasm	enzyme
*CP*	ceruloplasmin (ferroxidase)	27.838	Extracellular Space	enzyme
*EHD4*	EH-domain containing 4	2.361	Plasma Membrane	enzyme
GCLM	glutamate-cysteine ligase, modifier subunit	2.019	Cytoplasm	enzyme
HMOX1	heme oxygenase (decycling) 1	9.778	Cytoplasm	enzyme
MCM8	minichromosome maintenance complex component 8	2.027	Nucleus	enzyme
*MX1*	MX dynamin-like GTPase 1	28.177	Cytoplasm	enzyme
*PDE4B*	phosphodiesterase 4B, cAMP-specific	5.602	Cytoplasm	enzyme
RAB35	RAB35, member RAS oncogene family	2.086	Cytoplasm	enzyme
RRM2	ribonucleotide reductase M2	3.34	Nucleus	enzyme
*SDC1*	syndecan 1	13.681	Plasma Membrane	enzyme
*SETD8*	SET domain containing (lysine methyltransferase) 8	2.029	Nucleus	enzyme
SGPL1	sphingosine-1-phosphate lyase 1	3.108	Cytoplasm	enzyme
*SRXN1*	sulfiredoxin 1	6.306	Cytoplasm	enzyme
TGM2	transglutaminase 2	3.574	Cytoplasm	enzyme
*TOP2A*	topoisomerase (DNA) II alpha 170 kDa	2.26	Nucleus	enzyme
BCL11A	B-cell CLL/lymphoma 11A (zinc finger protein)	−2.38	Nucleus	transcription regulator
*BTG2*	BTG family, member 2	−2.22	Nucleus	transcription regulator
*CCAR1*	cell division cycle and apoptosis regulator 1	−2.943	Nucleus	transcription regulator
CSRNP1	cysteine-serine-rich nuclear protein 1	2.821	Nucleus	transcription regulator
*DEK*	DEK proto-oncogene	−3.006	Nucleus	transcription regulator
*DNAJB6*	DnaJ (Hsp40) homolog, subfamily B, member 6	−4.383	Nucleus	transcription regulator
ETV5	ets variant 5	−2.163	Nucleus	transcription regulator
*KLF13*	Kruppel-like factor 13	−2.006	Nucleus	transcription regulator
*KLF6*	Kruppel-like factor 6	6.003	Nucleus	transcription regulator
*NAA15*	N(alpha)-acetyltransferase 15, NatA auxiliary subunit	3.605	Nucleus	transcription regulator
*NFIX*	nuclear factor I/X (CCAAT-binding transcription factor)	−2.548	Nucleus	transcription regulator
NFKB2	nuclear factor of kappa light polypeptide gene enhancer in B-cells 2 (p49/p100)	2.768	Nucleus	transcription regulator
*PA2G4*	proliferation-associated 2G4, 38 kDa	−2.702	Nucleus	transcription regulator
PRDM2	PR domain containing 2, with ZNF domain	3.677	Nucleus	transcription regulator
*TBL1XR1*	transducin (beta)-like 1 X-linked receptor 1	2.587	Nucleus	transcription regulator
TCEB3	transcription elongation factor B (SIII), polypeptide 3 (110 kDa, elongin A)	3.053	Nucleus	transcription regulator
CAMK1G	calcium/calmodulin-dependent protein kinase IG	−2.271	Cytoplasm	kinase
*CAMK2N1*	calcium/calmodulin-dependent protein kinase II inhibitor 1	−11.813	Plasma Membrane	kinase
*CARD11*	caspase recruitment domain family, member 11	7.343	Cytoplasm	kinase
*CDK11A*	cyclin-dependent kinase 11A	−4.29	Nucleus	kinase
FGR	FGR proto-oncogene, Src family tyrosine kinase	3.915	Nucleus	kinase
HCK	HCK proto-oncogene, Src family tyrosine kinase	3.887	Cytoplasm	kinase
HSPB8	heat shock 22 kDa protein 8	4.112	Cytoplasm	kinase
LATS1	large tumor suppressor kinase 1	2.003	Nucleus	kinase
MAP4K4	mitogen-activated protein kinase kinase kinase kinase 4	2.258	Cytoplasm	kinase
NEK6	NIMA-related kinase 6	2.322	Nucleus	kinase
*SRPK2*	SRSF protein kinase 2	−5.614	Nucleus	kinase
TJP2	tight junction protein 2	2.552	Plasma Membrane	kinase
Ccl2	chemokine (C-C motif) ligand 2	195.455	Extracellular Space	cytokine
CCL3L3	chemokine (C-C motif) ligand 3-like 3	5.269	Extracellular Space	cytokine
CCL4	chemokine (C-C motif) ligand 4	2.162	Extracellular Space	cytokine
Ccl6	chemokine (C-C motif) ligand 6	10.291	Extracellular Space	cytokine
Ccl7	chemokine (C-C motif) ligand 7	124.78	Extracellular Space	cytokine
CXCL3	chemokine (C-X-C motif) ligand 3	13.211	Extracellular Space	cytokine
Cxcl9	chemokine (C-X-C motif) ligand 9	2.846	Extracellular Space	cytokine
*TIMP1*	TIMP metallopeptidase inhibitor 1	38.486	Extracellular Space	cytokine
IL6R	interleukin 6 receptor	2.315	Plasma Membrane	transmembrane receptor
*IL6ST*	interleukin 6 signal transducer	2.307	Plasma Membrane	transmembrane receptor
ITGB2	integrin, beta 2 (complement component 3 receptor 3 and 4 subunit)	2.675	Plasma Membrane	transmembrane receptor
TLR4	toll-like receptor 4	2.699	Plasma Membrane	transmembrane receptor
TNFRSF1A	tumor necrosis factor receptor superfamily, member 1A	3.555	Plasma Membrane	transmembrane receptor
UNC5B	unc-5 homolog B (C. elegans)	2.067	Plasma Membrane	transmembrane receptor
ALB	albumin	−3.125	Extracellular Space	transporter
*LCN2*	lipocalin 2	71.824	Extracellular Space	transporter
*RASA1*	RAS p21 protein activator (GTPase activating protein) 1	2.392	Cytoplasm	transporter
*TPR*	translocated promoter region, nuclear basket protein	2.212	Nucleus	transporter
*FGL2*	fibrinogen-like 2	16.793	Extracellular Space	peptidase
SENP2	SUMO1/sentrin/SMT3 specific peptidase 2	2.051	Nucleus	peptidase
DUSP5	dual specificity phosphatase 5	3.285	Nucleus	phosphatase
*PTPRF*	protein tyrosine phosphatase, receptor type, F	−6.365	Plasma Membrane	phosphatase
*EIF3C*	eukaryotic translation initiation factor 3, subunit C	−4.369	Other	translation regulator
*EIF5B*	eukaryotic translation initiation factor 5B	−3.044	Cytoplasm	translation regulator
FGF2	fibroblast growth factor 2 (basic)	2.387	Extracellular Space	growth factor
*KCND2*	potassium voltage-gated channel, Shal-related subfamily, member 2	−2.792	Plasma Membrane	ion channel
*AHI1*	Abelson helper integration site 1	2.243	Cytoplasm	other
BCL2A1	BCL2-related protein A1	3.055	Cytoplasm	other
CALB1	calbindin 1, 28 kDa	−2.091	Cytoplasm	other
*CD68*	CD68 molecule	4.365	Plasma Membrane	other
CDCA7L	cell division cycle associated 7-like	2.648	Nucleus	other
*CDT1*	chromatin licensing and DNA replication factor 1	3.098	Nucleus	other
*CISD2*	CDGSH iron sulfur domain 2	−7.833	Cytoplasm	other
*CMIP*	c-Maf inducing protein	−3.778	Cytoplasm	other
DAB2	Dab, mitogen-responsive phosphoprotein, homolog 2 (Drosophila)	3.053	Plasma Membrane	other
DES	desmin	2.857	Cytoplasm	other
DNAJB9	DnaJ (Hsp40) homolog, subfamily B, member 9	2.128	Nucleus	other
FLNA	filamin A, alpha	3.45	Cytoplasm	other
*GADD45G*	growth arrest and DNA-damage-inducible, gamma	3.191	Nucleus	other
*HLA-A*	major histocompatibility complex, class I, A	9.296	Plasma Membrane	other
HSPA2	heat shock 70 kDa protein 2	3.51	Cytoplasm	other
*LCP1*	lymphocyte cytosolic protein 1 (L-plastin)	6.082	Cytoplasm	other
*LSP1*	lymphocyte-specific protein 1	11.716	Cytoplasm	other
MMS22L	MMS22-like, DNA repair protein	2.918	Nucleus	other
MSI2	musashi RNA-binding protein 2	2.288	Cytoplasm	other
PDLIM7	PDZ and LIM domain 7 (enigma)	4.695	Cytoplasm	other
*PHLDA1*	pleckstrin homology-like domain, family A, member 1	5.129	Cytoplasm	other
*PMEPA1*	prostate transmembrane protein, androgen induced 1	2.682	Plasma Membrane	other
*PSIP1*	PC4 and SFRS1 interacting protein 1	−2.663	Nucleus	other
*RDX*	radixin	4.828	Cytoplasm	other
*SERPINA3*	serpin peptidase inhibitor, clade A (alpha-1 antiproteinase, antitrypsin), member 3	58.488	Extracellular Space	other
SPIN1	spindlin 1	2.178	Nucleus	other
SUDS3	suppressor of defective silencing 3 homolog (S. cerevisiae)	2.228	Nucleus	other
TAGLN2	transgelin 2	3.891	Cytoplasm	other
*THOC2*	THO complex 2	2.119	Nucleus	other
TMEM109	transmembrane protein 109	2.106	Cytoplasm	other
TMEM123	transmembrane protein 123	2.348	Plasma Membrane	other
*Orphan*				
*CYP1B1*	cytochrome P450, family 1, subfamily B, polypeptide 1	10.998	Cytoplasm	enzyme
*KIF3A*	kinesin family member 3A	−5.083	Cytoplasm	enzyme
PTGR1	prostaglandin reductase 1	2.258	Cytoplasm	enzyme
*RND3*	Rho family GTPase 3	2.864	Cytoplasm	enzyme
WFS1	Wolfram syndrome 1 (wolframin)	2.083	Cytoplasm	enzyme
ITPR2	inositol 1,4,5-trisphosphate receptor, type 2	2.489	Cytoplasm	ion channel
*KCNN4*	potassium intermediate/small conductance calcium-activated channel, subfamily N, member 4	3.088	Plasma Membrane	ion channel
*ATRX*	alpha thalassemia/mental retardation syndrome X-linked	2.091	Nucleus	transcription regulator
RAI14	retinoic acid induced 14	3.284	Nucleus	transcription regulator
CX3CL1	chemokine (C-X3-C motif) ligand 1	−2.044	Extracellular Space	cytokine
PTGER3	prostaglandin E receptor 3 (subtype EP3)	2.425	Plasma Membrane	G-protein coupled receptor
PI4K2A	phosphatidylinositol 4-kinase type 2 alpha	2.96	Cytoplasm	kinase
CD36	CD36 molecule (thrombospondin receptor)	5.08	Plasma Membrane	transmembrane receptor
ARL11	ADP-ribosylation factor-like 11	3.143	Other	other
*Brd4*	bromodomain containing 4	−3.528	Nucleus	other
CLN5	ceroid-lipofuscinosis, neuronal 5	2.041	Cytoplasm	other
*Ctdspl*	CTD (carboxy-terminal domain, RNA polymerase II, polypeptide A) small phosphatase-like	−7.271	Cytoplasm	other
*KIFAP3*	kinesin-associated protein 3	−2.281	Cytoplasm	other
*Nos1ap*	nitric oxide synthase 1 (neuronal) adaptor protein	−2.698	Other	other
PCDH15	protocadherin-related 15	2.147	Plasma Membrane	other
*RASSF4*	Ras association (RalGDS/AF-6) domain family member 4	4.289	Other	other
*Rbm25*	RNA binding motif protein 25	−5.547	Nucleus	other
*Slpi*	secretory leukocyte peptidase inhibitor	82.908	Cytoplasm	other
*Tpm3*	tropomyosin 3	2.592	Cytoplasm	other
*TRIM54*	tripartite motif containing 54	−4.426	Cytoplasm	other

Primary: >14 connections in GOI network (see text); Secondary: 8–14 connections in GOI network; Peripheral: <8 connections in GOI network; Orphan: No connections in GOI network; Italics= > Gene changes on both sides of the brain

**Fig. 9:**
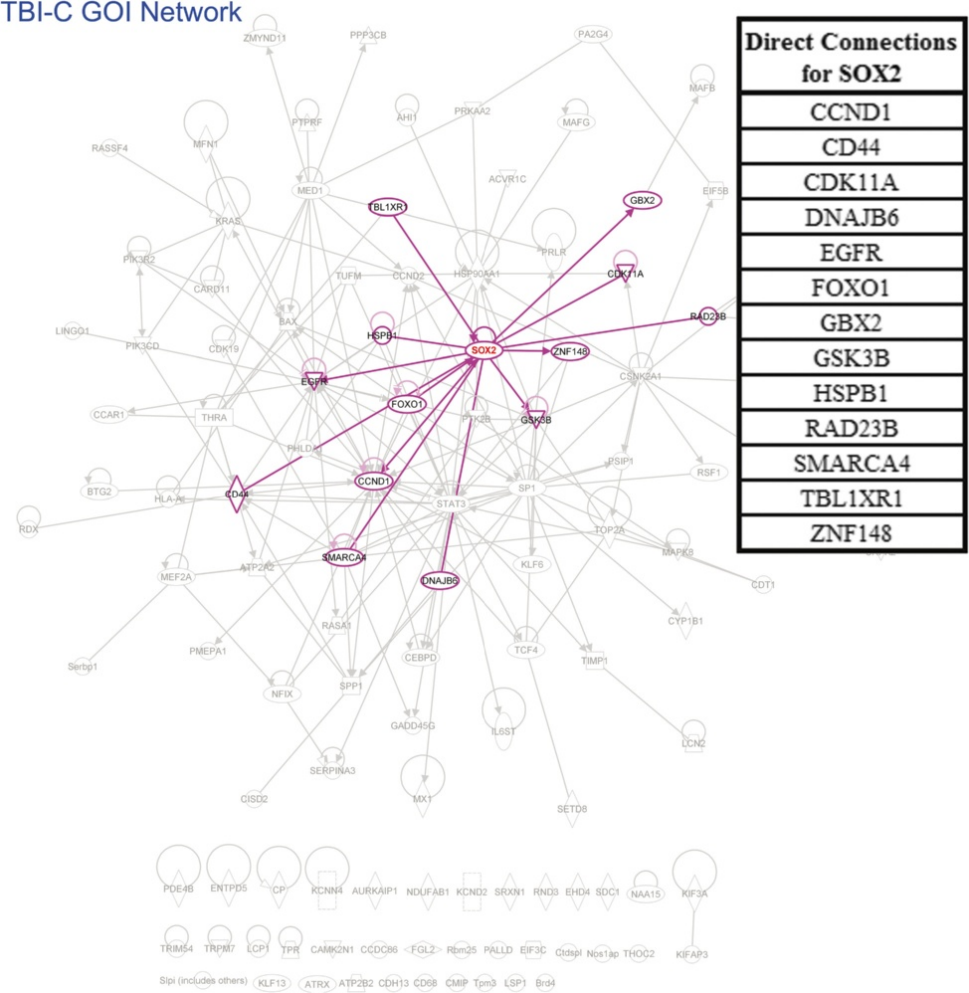
An example of calculating the number of direct connections for the TBI-C GOI network. In IPA, the gene in question was selected (SOX2 in this example). Then, its direct connections were selected by right clicking on SOX2 and using the “select nearest neighbors” option (highlighted in blue). A list of the selected genes was exported and SOX2 was removed from the list (upper right corner). The remaining genes were counted (13 in this example) and SOX2 was ranked in the TBI-C gene interaction hierarchy (primary tier) by this number

TBI-C: A total of 115 GOI were identified. Our analysis showed that 78 of the GOI formed an interconnected network, leaving 37 “orphan” genes (see Additional file 5). Genes having 1st order connections with more than 10 % of the other genes within the main GOI network (>8 connections) were considered “primary” in this analysis (see Fig. 9 for an example). Genes having connections with 5 %–10 % of the other genes (4–8 connections) were considered “secondary” (see Additional file 6 for an example) and those with connections with less than 5 % of the other genes (<4 connections) were considered “peripheral”. The resultant GIH is displayed in Table 7.

**Table 7:** TBI-C Gene interaction hierarchy (GIH)

Gene symbol	Entrez gene name	Fold change	Cellular compartment	Molecular type
*Primary*				
*CCND1*	cyclin D1	−2.027	Nucleus	transcription regulator
MED1	mediator complex subunit 1	−4.011	Nucleus	transcription regulator
*SMARCA4*	SWI/SNF related, matrix associated, actin dependent regulator of chromatin, subfamily a, member 4	−7.712	Nucleus	transcription regulator
SOX2	SRY (sex determining region Y)-box 2	−4.791	Nucleus	transcription regulator
SP1	Sp1 transcription factor	−2.076	Nucleus	transcription regulator
*STAT3*	signal transducer and activator of transcription 3 (acute-phase response factor)	−3.771	Nucleus	transcription regulator
*CSNK2A1*	casein kinase 2, alpha 1 polypeptide	−2.75	Cytoplasm	kinase
*EGFR*	epidermal growth factor receptor	2.374	Plasma Membrane	kinase
*GSK3B*	glycogen synthase kinase 3 beta	−6.635	Nucleus	kinase
*CD44*	CD44 molecule (Indian blood group)	2.399	Plasma Membrane	enzyme
HSP90AA1	heat shock protein 90 kDa alpha (cytosolic), class A member 1	−4.843	Cytoplasm	enzyme
*Secondary*				
FOXO1	forkhead box O1	−3.329	Nucleus	transcription regulator
MEF2A	myocyte enhancer factor 2A	−6.31	Nucleus	transcription regulator
*NFIX*	nuclear factor I/X (CCAAT-binding transcription factor)	−8.112	Nucleus	transcription regulator
*TCF4*	transcription factor 4	−4.625	Nucleus	transcription regulator
MAPK8	mitogen-activated protein kinase 8	2.102	Cytoplasm	kinase
PIK3R2	phosphoinositide-3-kinase, regulatory subunit 2 (beta)	2.332	Cytoplasm	kinase
PTK2B	protein tyrosine kinase 2 beta	2.15	Cytoplasm	kinase
KRAS	Kirsten rat sarcoma viral oncogene homolog	−2.027	Cytoplasm	enzyme
*TOP2A*	topoisomerase (DNA) II alpha 170 kDa	−2.406	Nucleus	enzyme
ATP2A2	ATPase, Ca++ transporting, cardiac muscle, slow twitch 2	−2.607	Cytoplasm	transporter
BAX	BCL2-associated X protein	−3.306	Cytoplasm	transporter
*SPP1*	secreted phosphoprotein 1	2.37	Extracellular Space	cytokine
*THRA*	thyroid hormone receptor, alpha	−11.518	Nucleus	ligand-dependent nuclear receptor
TUFM	Tu translation elongation factor, mitochondrial	−2.109	Cytoplasm	translation regulator
CCND2	cyclin D2	−3.617	Nucleus	other
*HSPB1*	heat shock 27 kDa protein 1	2.639	Cytoplasm	other
*Peripheral*				
*BTG2*	BTG family, member 2	−5.803	Nucleus	transcription regulator
*CCAR1*	cell division cycle and apoptosis regulator 1	−11.648	Nucleus	transcription regulator
*CEBPD*	CCAAT/enhancer binding protein (C/EBP), delta	2.037	Nucleus	transcription regulator
*DEK*	DEK proto-oncogene	−7.352	Nucleus	transcription regulator
*DNAJB6*	DnaJ (Hsp40) homolog, subfamily B, member 6	5.614	Nucleus	transcription regulator
GBX2	gastrulation brain homeobox 2	2.59	Nucleus	transcription regulator
*KLF6*	Kruppel-like factor 6	2.865	Nucleus	transcription regulator
MAFG	v-maf avian musculoaponeurotic fibrosarcoma oncogene homolog G	−2.632	Nucleus	transcription regulator
MTDH	metadherin	−2.544	Cytoplasm	transcription regulator
*PA2G4*	proliferation-associated 2G4, 38 kDa	−5.783	Nucleus	transcription regulator
RSF1	remodeling and spacing factor 1	−2.618	Nucleus	transcription regulator
*TBL1XR1*	transducin (beta)-like 1 X-linked receptor 1	−2.134	Nucleus	transcription regulator
ZMYND11	zinc finger, MYND-type containing 11	−2.211	Nucleus	transcription regulator
ZNF148	zinc finger protein 148	2.114	Nucleus	transcription regulator
ACIN1	apoptotic chromatin condensation inducer 1	−2.515	Nucleus	enzyme
*CYP1B1*	cytochrome P450, family 1, subfamily B, polypeptide 1	4.808	Cytoplasm	enzyme
DPYD	dihydropyrimidine dehydrogenase	2.292	Cytoplasm	enzyme
MFN1	mitofusin 1	2.304	Cytoplasm	enzyme
*MX1*	MX dynamin-like GTPase 1	7.326	Cytoplasm	enzyme
*SETD8*	SET domain containing (lysine methyltransferase) 8	−3.93	Nucleus	enzyme
TTLL1	tubulin tyrosine ligase-like family, member 1	2.284	Extracellular Space	enzyme
ACVR1C	activin A receptor, type IC	−9.107	Plasma Membrane	kinase
*CARD11*	caspase recruitment domain family, member 11	2.892	Cytoplasm	kinase
*CDK11A*	cyclin-dependent kinase 11A	−14.872	Nucleus	kinase
CDK19	cyclin-dependent kinase 19	−2.191	Nucleus	kinase
PIK3CD	phosphatidylinositol-4,5-bisphosphate 3-kinase, catalytic subunit delta	−2.113	Cytoplasm	kinase
PRKAA2	protein kinase, AMP-activated, alpha 2 catalytic subunit	−2.546	Cytoplasm	kinase
*SRPK2*	SRSF protein kinase 2	−23.589	Nucleus	kinase
PPP3CB	protein phosphatase 3, catalytic subunit, beta isozyme	2.1	Plasma Membrane	phosphatase
*PTPRF*	protein tyrosine phosphatase, receptor type, F	−20.492	Plasma Membrane	phosphatase
*IL6ST*	interleukin 6 signal transducer	−3.283	Plasma Membrane	transmembrane receptor
PRLR	prolactin receptor	−3.192	Plasma Membrane	transmembrane receptor
*LCN2*	lipocalin 2	3.895	Extracellular Space	transporter
*RASA1*	RAS p21 protein activator (GTPase activating protein) 1	−2.105	Cytoplasm	transporter
*TIMP1*	TIMP metallopeptidase inhibitor 1	2.101	Extracellular Space	cytokine
*EIF5B*	eukaryotic translation initiation factor 5B	−8.766	Cytoplasm	translation regulator
*AHI1*	Abelson helper integration site 1	−2.897	Cytoplasm	other
*CDT1*	chromatin licensing and DNA replication factor 1	−2.295	Nucleus	other
*CISD2*	CDGSH iron sulfur domain 2	−19.012	Cytoplasm	other
*GADD45G*	growth arrest and DNA-damage-inducible, gamma	−2.384	Nucleus	other
*HLA-A*	major histocompatibility complex, class I, A	3.657	Plasma Membrane	other
LINGO1	leucine rich repeat and Ig domain containing 1	−2.173	Plasma Membrane	other
MAFB	v-maf avian musculoaponeurotic fibrosarcoma oncogene homolog B	−2.018	Nucleus	other
*PHLDA1*	pleckstrin homology-like domain, family A, member 1	2.16	Cytoplasm	other
*PMEPA1*	prostate transmembrane protein, androgen induced 1	−2.937	Plasma Membrane	other
*PSIP1*	PC4 and SFRS1 interacting protein 1	2.113	Nucleus	other
RAD23B	RAD23 homolog B (S. cerevisiae)	−2.217	Nucleus	other
*RASSF4*	Ras association (RalGDS/AF-6) domain family member 4	2.106	Other	other
*RDX*	radixin	−5.274	Cytoplasm	other
Serbp1	Serpine1 mRNA binding protein 1	−2.059	Cytoplasm	other
*SERPINA3*	serpin peptidase inhibitor, clade A (alpha-1 antiproteinase, antitrypsin), member 3	2.509	Extracellular Space	other
*Orphan*				
AURKAIP1	aurora kinase A interacting protein 1	−2.023	Nucleus	enzyme
*CP*	ceruloplasmin (ferroxidase)	8.477	Extracellular Space	enzyme
*EHD4*	EH-domain containing 4	−2.056	Plasma Membrane	enzyme
ENTPD5	ectonucleoside triphosphate diphosphohydrolase 5	−2.055	Cytoplasm	enzyme
*KIF3A*	kinesin family member 3A	−11.754	Cytoplasm	enzyme
NDUFAB1	NADH dehydrogenase (ubiquinone) 1, alpha/beta subcomplex, 1, 8 kDa	−2.028	Cytoplasm	enzyme
*PDE4B*	phosphodiesterase 4B, cAMP-specific	2.359	Cytoplasm	enzyme
*RND3*	Rho family GTPase 3	−2.971	Cytoplasm	enzyme
*SDC1*	syndecan 1	2.566	Plasma Membrane	enzyme
*SRXN1*	sulfiredoxin 1	2.402	Cytoplasm	enzyme
*ATRX*	alpha thalassemia/mental retardation syndrome X-linked	−5.964	Nucleus	transcription regulator
*KLF13*	Kruppel-like factor 13	−4.582	Nucleus	transcription regulator
*NAA15*	N(alpha)-acetyltransferase 15, NatA auxiliary subunit	−3.751	Nucleus	transcription regulator
*KCND2*	potassium voltage-gated channel, Shal-related subfamily, member 2	−7.585	Plasma Membrane	ion channel
*KCNN4*	potassium intermediate/small conductance calcium-activated channel, subfamily N, member 4	−9.429	Plasma Membrane	ion channel
*CAMK2N1*	calcium/calmodulin-dependent protein kinase II inhibitor 1	−23.824	Plasma Membrane	kinase
TRPM7	transient receptor potential cation channel, subfamily M, member 7	2.226	Plasma Membrane	kinase
ATP2B2	ATPase, Ca++ transporting, plasma membrane 2	2.276	Plasma Membrane	transporter
*TPR*	translocated promoter region, nuclear basket protein	−2.728	Nucleus	transporter
*FGL2*	fibrinogen-like 2	4.017	Extracellular Space	peptidase
*EIF3C*	eukaryotic translation initiation factor 3, subunit C	−9.072	Other	translation regulator
*Brd4*	bromodomain containing 4	−15.202	Nucleus	other
CCDC86	coiled-coil domain containing 86	−2.149	Nucleus	other
*CD68*	CD68 molecule	2.007	Plasma Membrane	other
CDH13	cadherin 13	−2.692	Plasma Membrane	other
*CMIP*	c-Maf inducing protein	−13.763	Cytoplasm	other
*Ctdspl*	CTD (carboxy-terminal domain, RNA polymerase II, polypeptide A) small phosphatase-like	−36.886	Cytoplasm	other
*KIFAP3*	kinesin-associated protein 3	−7.831	Cytoplasm	other
*LCP1*	lymphocyte cytosolic protein 1 (L-plastin)	2.799	Cytoplasm	other
*LSP1*	lymphocyte-specific protein 1	2.14	Cytoplasm	other
*Nos1ap*	nitric oxide synthase 1 (neuronal) adaptor protein	−5.717	Other	other
PALLD	palladin, cytoskeletal associated protein	−5.086	Plasma Membrane	other
*Rbm25*	RNA binding motif protein 25	−16.213	Nucleus	other
*Slpi*	secretory leukocyte peptidase inhibitor	3.119	Cytoplasm	other
*THOC2*	THO complex 2	−4.886	Nucleus	other
*Tpm3*	tropomyosin 3	−2.715	Cytoplasm	other
*TRIM54*	tripartite motif containing 54	−2.032	Cytoplasm	other

Primary: >8 connections in GOI network (see text); Secondary: 4–8 connections in GOI network; Peripheral: <4 connections in GOI network; Orphan: No connections in GOI network; Italics= > Gene changes on both sides of the brain

### Cell cycle genes included in the GIHs

**Fig. 10:**
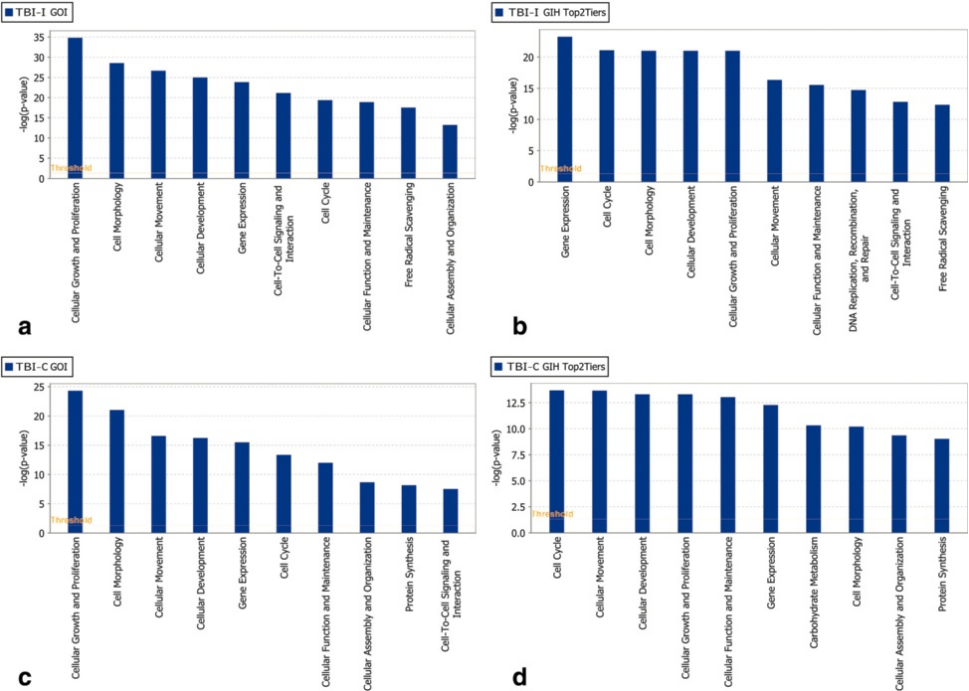
Functional analysis of GOI and top 2 GIH tiers. The top 10 molecular and cellular functions determined by IPA to be associated with the unranked GOI for TBI-I (**a**) and TBI-C (**c**) and the primary and secondary tiers of the TBI-I (**b**) and TBI-C (**d**) GIHs. Side by side comparison allowed for visualization of how functions changed in significance order once the genes were put into a ranked order. Notably, cell cycle moved up to be ranked second on both sides of the brain. *The cell death and survival category was removed from this analysis because all genes were initially selected from that functional category*

We performed an IPA molecular and cellular functional analysis on the unranked GOI for both datasets and the top 2 tiers (most significant by our definition) of our resultant GIHs to further elucidate the most significant biological functions post-TBI (Fig. 10). The cell death and survival category was removed from this analysis since all genes were initially selected from this functional category. When analyzing the top 2 tiers of the GIHs, cell cycle was ranked second for TBI-I and first for TBI-C. Is was also the highest ranked molecular and cellular function common to both sides (Fig. 10b, d). The cell cycle moved up 5 functional ranking spots on both sides of the brain from where it was ranked prior to the GIH analysis. This result was intriguing because aberrant attempts to reactivate the cell cycle by post-mitotic neurons have been implicated as a trigger for apoptosis [26, 27]. By cross-referencing our GIHs with genes that IPA includes in the cell cycle upper level biological function, we determined that 74 genes in the TBI-I GIH and 47 genes in the TBI-C GIH were associated with the cell cycle (Tables 8 and 9). Just over 85 % of the cell cycle genes increased in expression ipsilaterally compared to controls. The relative inverse is true contralaterally with nearly 79 % of the cell cycle genes decreasing in expression. Remarkably, 83 % of TBI-I and 70 % of TBI-C primary and secondary tier genes were classified as cell cycle genes (TBI-I: 35 of 42 genes; TBI-C: 19 of 27 genes).

**Table 8:** Cell cycle genes in the TBI-I gene interaction hierarchy by tier

Primary	Secondary	Peripheral		Orphan
ATF3	BAG3	BCL2A1	NEK6	ATRX
CASP3	CASP7	BTG2	NFIX	Brd4
CCND1	CCNA2	CAMK2N1	PA2G4	CYP1B1
CD44	CDKN1B	Ccl2	PDLIM7	
CDK1	CEBPD	CDK11A	PMEPA1	
CEBPB	CREM	CDT1	PRDM2	
CREB1	FOSL1	DEK	PTPRF	
CREBBP	HSPA1A/HSPA1B	ETV5	RAB35	
CSNK2A1	HSPB1	FGF2	SETD8	
EGFR	IKBKB	FLNA	SRPK2	
ELAVL1	IL1B	GADD45G	SUDS3	
FN1	KLF4	HMOX1	TBL1XR1	
GSK3B	KPNB1	HSPA2	TCEB3	
MDM2	MCL1	IL6R	THOC2	
NFE2L2	MCM2	KLF6	TIMP1	
SMARCA4	MITF	LATS1	TNFRSF1A	
STAT3	PTGS2	MCM8	TOP2A	
	SPP1	MMS22L	TPR

**Table 9:** Cell cycle genes in the TBI-C gene interaction hierarchy by tier

Primary	Secondary	Peripheral		Orphan
CCND1	BAX	ACIN1	MTDH	ATRX
CD44	CCND2	BTG2	PA2G4	Brd4
CSNK2A1	FOXO1	CDK11A	PMEPA1	CAMK2N1
EGFR	HSPB1	CDK19	PRKAA2	CDH13
GSK3B	KRAS	CDT1	PTPRF	ENTPD5
SMARCA4	MAPK8	CEBPD	RSF1	THOC2
SOX2	NFIX	CYP1B1	SETD8	TPR
SP1	PTK2B	DEK	SRPK2	
STAT3	SPP1	GADD45G	TBL1XR1	
	TOP2A	KLF6	TIMP1	
		MAFB		

### Real-time PCR

**Fig. 11:**
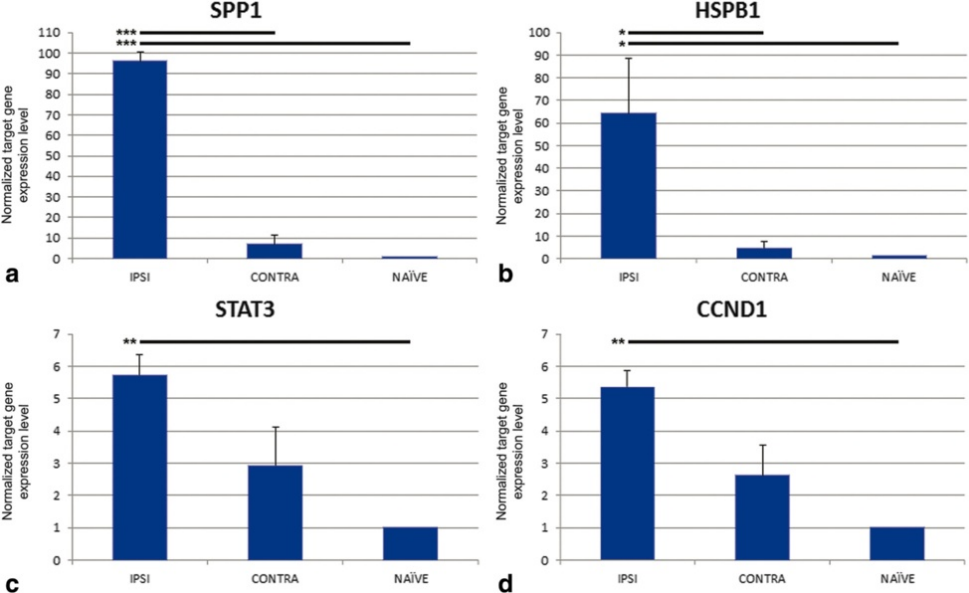
Real-time PCR results for selected genes. SPP1, HSPB1, STAT3, and CCND1 were chosen for real-time PCR studies. Using the ΔΔCt method, the normalized target gene expression level was given by 2^-ΔΔCt^. For all genes, ipsilateral (IPSI) expression was significantly different from naïve (**a**-**d**). Ipsilateral expression was also significantly different from contralateral (CONTRA) expression for SPP1 (**a**) and HSPB1 (**b**). The comparison of ipsilateral to contralateral expression for STAT3 (**c**; *p =* 0.088) and CCND1 (**d**; *p =* 0.063) fell short of statistical significance. Contralateral expression was not significantly different from naïve for any genes. The results are shown as mean ± SE. * p < 0.05, ** p < 0.01, *** p < 0.005

As expected, ipsilateral expression was significantly increased compared to naïve for all genes tested following TBI (Fig. 11). However, ipsilateral expression was only significantly different from contralateral expression for SPP1 and HSPB1 while this comparison for STAT3 (*p =* 0.088) and CCND1 (*p =* 0.063) fell short of statistical significance. Contralateral expression was not significantly different from naïve for any of the genes tested.

## Discussion

We used microarray technology and subsequent bioinformatic analysis in this study to examine molecular and functional alterations following TBI. Not surprisingly, cell death and survival was determined to be a significant molecular and cellular function associated with the genes expressed ipsilateral to the injury. Interestingly, while cell death was not observed on the contralateral side of the brain, there was significant modulation of cell death and survival genes and this molecular and cellular function is very highly associated with the gene expression pattern.

Our histology results using markers for cell damage (FJB) and DNA fragmentation (TUNEL) suggest a potential opportunity for therapeutic intervention. At 24 h post-injury, there is a developing cortical cavity at the site of impact surrounded with FJB and TUNEL-positive cells. Therapy aimed at preserving cortical tissue should be administered in the acute period to exert maximal neuroprotective effects. However, while there is significant correlation between FJB and TUNEL staining in the cortex at this time point, no TUNEL-positive cells were detected in the hippocampus where FJB detected some neuronal damage on the ipsilateral side. Similar histology results were recently seen with our model of nerve agent exposure [20] and a neuroprotective agent was able to rescue the hippocampal neurons [28]. This suggests that these hippocampal neurons have not yet progressed to the point of apoptosis and an extended therapeutic window may exist for subcortical brain areas.

Our microarray data showed that TBI resulted in significant alterations in CD gene expression on both sides of the brain. Nearly 45 % of the differentially expressed CD genes were common to both sides of the brain and 82 % of those genes changed similarly. However, a distinct expression pattern was exhibited by the balance of the common CD genes and those that change in expression uniquely on one side of the brain. The vast majority of these ipsilateral CD genes increased in expression compared to controls, while the majority of these contralateral CD genes decreased in expression compared to controls or were reduced compared to ipsilateral expression. Notable was the expression of key apoptosis-related genes. BCL2A1, caspases 3 and 7, CDK1, cyclins A2 and D1, and NFKB2 showed increased expression ipsilaterally, while BAX, cyclins D1and D2, KRAS and PIK3CD showed decreased expression contralaterally.

It is important to note here that the real-time PCR results for the genes selected did not agree totally with the microarray results. This was especially true for the contralateral samples. However, it has been shown that the correlation between microarray and real-time PCR results is lower for genes showing decreased expression and having lower fold changes [29]. Our results do show better correlation with the large TBI-I fold change genes SPP1 (37.9 fold) and HSPB1 (46.9 fold). The remaining fold changes for the selected genes are less than ± 4.22 with most in the 2.0-2.6 range. While further validation including more genes and a larger sample size may be needed for subsequent studies, these PCR results are consistent with expression of these genes being higher for TBI-I and lower for TBI-C. It is in this context that the discussion of the microarray results continues.

As stated above, this contralateral expression pattern in our model may indicate an endogenous effort to suppress cell death promoting genes remote from the injury in order to prevent spreading of the injury and offer additional protection from additional insults, similar to gene expression changes in ischemic preconditioning [30, 31]. An analogous and potentially neuroprotective gene expression pattern was observed in an *in vitro* model of mild TBI where the modulation of genes reflected an endogenous effort to prevent oxidative/nitrosative stress and apoptosis during a transient period of mitochondrial malfunctioning [32]. We have previously reported a similar gene expression pattern for inflammatory response genes following TBI [12]. In that previous study, genes from both sides of the brain were pooled for analysis. Because we now believe that analyzing gene expression on the contralateral side is critical to understanding endogenous protective mechanisms, the full GIH analysis [33] was performed on each side of the brain separately. By determining the key molecules involved in the endogenous effort to suppress cell death, it may be possible to develop molecular strategies to provide neuroprotection for the injured brain as well as augment the endogenous neuroprotective process.

We identified 170 TBI-I and 115 TBI-C GOI through canonical pathway and network analysis combined with the common genes that change differently on each side of the brain. Many of these genes have been previously associated with acute brain injuries (i.e., TBI, stroke) but not all of them have been connected to the cell death caused by these injuries. These genes include BAX, CASP3, CCNA2, CCND1, CD44, CD68, CEBPD, GSK3B, HSPB1, IL1B, LCN2, NFKB2, SERPINA3, SPP1, STAT3, TIMP1, TNFRSF1A, and TOP2A [14, 34–42]. This supported the idea that our methods for identifying genes of interest targets important genes in the post-injury response. Several genes which have been linked to cell death in cancer, epilepsy, or psychological disorders but not yet associated with brain injury, including CSNK2A1, ELAVL1, MITF, and SMARCA4, were also identified which may provide additional therapeutic targets for prevention of cell death following TBI. We next wanted to determine which genes were central to cell death processes. We approached this by creating a network of our GOI within IPA and determining how many 1st order connections each gene had with the other genes in the network. A GIH was created based on these numbers and distinct patterns in terms of molecular type were found.

For TBI-I, transcription regulators were the predominant molecular type in the top 2 tiers of the GIH. This result was expected from our previous GIH analyses [12]. After the transcription regulators, kinases and unspecified enzymes were prominent in the top 2 tiers of the TBI-I GIH. In the peripheral tier, unspecified enzymes, transcription regulators and kinases were most represented. Cytokines, transmembrane receptors, and transporters also had notable numbers in the peripheral tier. Remarkably, only 2 cytokines, IL1B and SPP1, are included in the top 2 tiers of this GIH. This result is not unexpected as previous GIH analysis had shown that the near 1-to-1 relationship that cytokines have with their receptors limits the 1st order connections these molecules have in the GOI network [12].

Transcription regulators were also predominant in the top 2 tiers of the TBI-C GIH followed by kinases and unspecified enzymes. These same molecular types headed the peripheral tier as well with transcription regulators ahead of enzymes and kinases. Other notable molecular types in the peripheral tier were phosphatases, transmembrane receptors, and transporters. Again, cytokines do not have significant numbers in this GIH. Our analysis strongly suggests that other molecular types, transcription regulators, kinases, and other enzymes in this case, may be better therapeutic targets because they have the potential to impact the overall cell death process to a greater extent.

Very intriguing in our cell death analysis was how cell cycle moved up significantly in functional ranking on both sides of the brain when comparing the functional analysis for unranked GOI to that for the top 2 tiers of our GIHs. Cell cycle molecules have be implicated as apoptotic mediators for post-mitotic cells under stress due to trauma or neurological disease. It is believed that there is an aberrant attempt the re-enter the cell cycle that causes the cells to eventually undergo apoptosis [26, 43–48]. Much attention has been given to the cyclin-dependent kinases (CDKs), cyclins, which activate the CDKs [27, 48, 49], and CDK inhibitors. Significant evidence for CDK involvement in cell cycle-related apoptosis has come from the experimental use of exogenous CDK inhibitors that prevented apoptosis [47, 50–56]. Pertinent to this discussion, evidence has shown that CDK1, when activated by cyclin A [57], and CDK4 and CDK6, when activated by cyclin D in post-mitotic neurons, can lead to cell death via caspase-dependent apoptosis [26, 27, 44, 49]. Additionally, ablation of cyclin D1 reduces neurodegeneration caused by TBI [58]. CDK11 has been shown to initiate apoptosis by interacting with either cyclin D3 [59] or eukaryotic translation initiation factor 3 subunit F (EIF3F) [60]. In our model, cyclins A2 and D1 are increased ipsilaterally, consistent with other studies [27, 47, 50], while both cyclins D1 and D2 are decreased contralaterally. CDK1 and the CDK4 inhibitors, CDKN1A (p21,Cip1 (not in GIH)) and CDKN1B (p27,Kip1), are all increased ipsilaterally. CDK11 (CDK11A (both sides); CDK19 (TBI-C only)) decreases in expression on both sides of the brain. While not found in our analysis, EIF3F is part of the functional core of EIF along with EIF3A (TBI-C only (not in GIH)) and EIF3C (both sides) which decrease in expression following TBI [61]. It is plausible that apoptosis would occur in this injury state because these molecules are not being expressed in the tightly controlled manner necessary to properly navigate the cell cycle [46, 55]. Other CDKs have also been implicated in apoptosis and excitotoxic cell death [26, 49, 51, 52, 62, 63] but our GIH does not point to those as major players.

In addition to 4 TBI-I and 2 TBI-C CDK-related genes, IPA classified 31 other TBI-I genes and 17 other TBI-C genes in the top 2 tiers of their respective GIHs as cell cycle genes. It should be noted that cell cycle is an upper level function in IPA. That means these genes, while associated with the cell cycle, are not necessarily integral to its progression. These genes fell into 3 general categories. The first category included those genes that have been experimentally linked to a model of TBI. Genes in this category were ATF3, BAG3, CASP3, CASP7, CD44, CEBPB, CEBPD, CREB1, CREM, EGFR, FN1, FOSL1, GSK3B, HSPA1A/HSPA1B, HSPB1, IKBKB, IL1B, KLF4, MCL1, MDM2, NFE2L2, PTGS2, SPP1, and STAT3 for TBI-I [22, 36, 40–42, 64–78] and BAX, CD44, EGFR, FOXO1, GSK3B, HSPB1, MAPK8, SOX2, SPP1, and STAT3 for TBI-C [22, 36, 40, 41, 67, 71, 78–81]. The second category included genes that had been observed in models of hypoxia/ischemia, chemical brain lesions, or spinal cord injury. Genes in this category were CREBBP and KPNB1 for TBI-I [82, 83] and KRAS, PTK2B, SP1, and TOP2A for TBI-C [84–87]. The third category included genes that were previously linked only to the progression of cancers or psychotic disorders and, therefore, novel to a discussion of cell death following TBI. Genes in this category were CSNK2A1, ELAVL1, MCM2, MITF, and SMARCA4 for TBI-I and CSNK2A1, NFIX, and SMARCA4 for TBI-C. The specifics of how these genes are associated with the cell cycle and affect cell death are beyond the scope of this analysis. However, our GIH analysis would suggest that these genes would be intriguing targets for further study in relation to post-TBI cell death. Specifically, CCND1, CSNK2A1, SMARCA4, and STAT3 were included in the top 2 tiers for both datasets and exhibit increased expression in TBI-I and decreased expression in TBI-C. Additionally, cyclin D2 and 2 apoptosis signaling genes, BAX and KRAS, are in the secondary tier of the TBI-C GIH and show decreased expression. Targeting these key molecules showing contralateral suppression for potential therapies may prove effective because their expression correlates to the observed absence of cell death.

## Conclusions

Unilateral TBI results in significant gene expression changes on both sides of the brain. The overall gene expression pattern in the brain suggests a suppression of CD genes contralateral to the injury which may be an endogenous protective mechanism. Using canonical pathways and IPA generated networks as a guide, we were able to identify genes that were central to the post-TBI CD gene response. Further network analysis allowed for the ranking of these genes into GIHs. The GIH ranking then led to the identification of cell cycle as a key molecular and cellular function on both sides of the brain. Significantly, several cell cycle molecules were identified in this analysis that exhibit increased expression ipsilaterally and decreased expression contralaterally. GIH analysis relies on connections in a virtual network. Future experiments will use discrete microdissected portions of the brain (cortex, hippocampus, striatum) in order to increase the likelihood that the molecular interactions described in the network actually do occur *in vivo*. This will increase the power of the GIH analysis. Further real-time PCR confirmation will be necessary with an emphasis on contralateral and decreased gene expression. Also, proteomic confirmation will be necessary to show that *in vivo* protein levels match our microarray results [88, 89]. Once confirmed, the key CD molecules suggested by our GIH can be further explored. Additional exploration into the remote suppression of CD genes may provide insight into neuroprotective mechanisms that could be used to develop therapies to prevent cell death following TBI.

## Additional files

Additional file 1:
**Examples of** **TBI-I** **networks.** TBI-I CD networks 1 (A), 3 (B), 5 (C), and 6 (D) (see Table 2) with all gene families, groups and complexes expanded to show the member genes and showing the relative expression values of potential GOI for TBI-I. *red: relative increase in expression; green: relative decrease in expression; white: no change in expression; gold connections and outlines: expansion of gene families, groups and complexes in the original network. (TIF 4.06 mb)*
Additional file 2:
**Examples of** **TBI-C** **networks.** TBI-C CD networks 1 (A), 3 (B), 5 (C), and 6 (D) (see Table 3) with all gene families, groups and complexes expanded to show the member genes and showing the relative expression values of potential GOI for TBI-C. *red: relative increase in expression; green: relative decrease in expression; white: no change in expression; gold connections and outlines: expansion of gene families, groups and complexes in the original network. (TIF 4.30 mb)*
Additional file 3:
**The** **TBI-I** **GOI network.** This is the resultant network when IPA connected our 170 TBI-I GOI using only direct (1st order) connections between the genes. 145 of the GOI formed an interconnected network, leaving 25 “orphan” genes. (TIF 4.20 mb) Additional file 4:
**An example of calculating the number of direct connections for the** **TBI-I** **GOI network.** In IPA, the gene in question was selected (HSPB1 in this example). Then, its direct connections were selected by right clicking on HSPB1 and using the “select nearest neighbors” option (highlighted in purple). A list of the selected genes was exported and HSPB1 was removed from the list (upper right corner). The remaining genes were counted (11 in this example) and HSPB1 was ranked in the TBI-I gene interaction hierarchy (secondary tier) by this number. (TIF 3.99 mb) Additional file 5:
**The** **TBI-C** **GOI network.** This is the resultant network when IPA connected our 115 TBI-C GOI using only direct (1st order) connections between the genes. 78 of the GOI formed an interconnected network, leaving 37 “orphan” genes. (TIF 4.84 mb) Additional file 6:
**An example of calculating the number of direct connections for the** **TBI-C** **GOI network.** In IPA, the gene in question was selected (CCND2 in this example). Then, its direct connections were selected by right clicking on CCND2 and using the “select nearest neighbors” option (highlighted in purple). A list of the selected genes was exported and CCND2 was removed from the list (upper right corner). The remaining genes were counted (6 in this example) and CCND2 was ranked in the TBI-C gene interaction hierarchy (secondary tier) by this number. (TIF 4.69 mb)

## Abbreviations

CCI: controlled cortical impact
FJB: fluoro-Jade B
GIH: gene interaction hierarchy
GOI: genes of interest
IPA: ingenuity pathway analysis
CD: cell death and survival
TBI: traumatic brain injury
TBI-C: contralateral vs naïve gene dataset
TBI-I: ipsilateral vs naïve gene dataset
TUNEL: terminal deoxynucleotidyl transferase dUTP nick end labeling
