# Supplementary figures and images for: Bilateral gene interaction hierarchy analysis of the cell death gene response emphasizes the significance of cell cycle genes following unilateral traumatic brain injury
Source: BMC Genomics. 2016 Feb 24;17:130. doi: 10.1186/s12864-016-2412-0 (PMC4765060; doi:10.1186/s12864-016-2412-0)

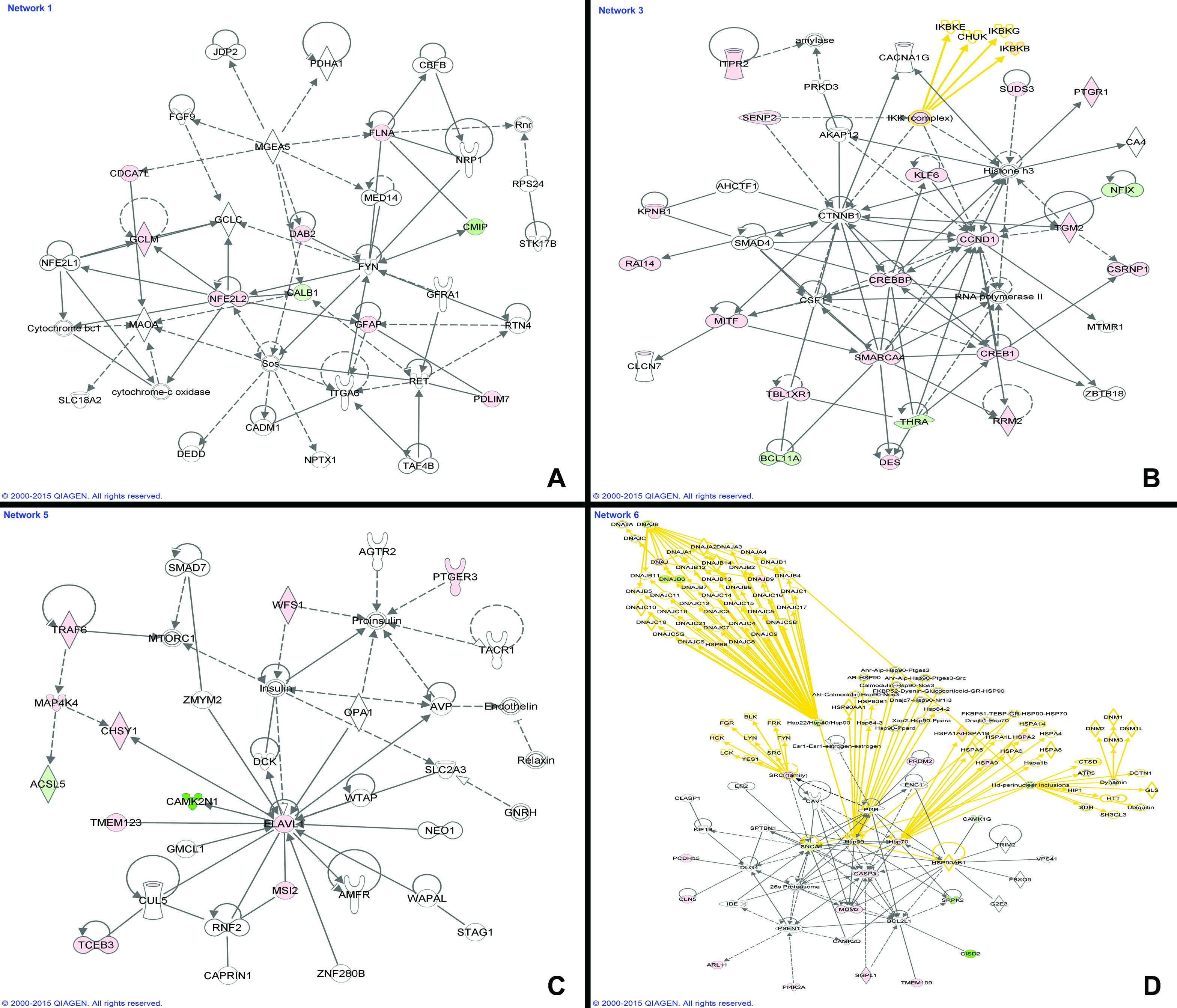

Supplement: Additional file 1: — Examples of TBI-I networks. TBI-I CD networks 1 (A), 3 (B), 5 (C), and 6 (D) (see Table 2) with all gene families, groups and complexes expanded to show the member genes and showing the relative expression values of potential GOI for TBI-I. red: relative increase in expression; green: relative decrease in expression; white: no change in expression; gold connections and outlines: expansion of gene families, groups and complexes in the original network. (TIF 4.06 mb) [file 12864_2016_2412_MOESM1_ESM.tif]

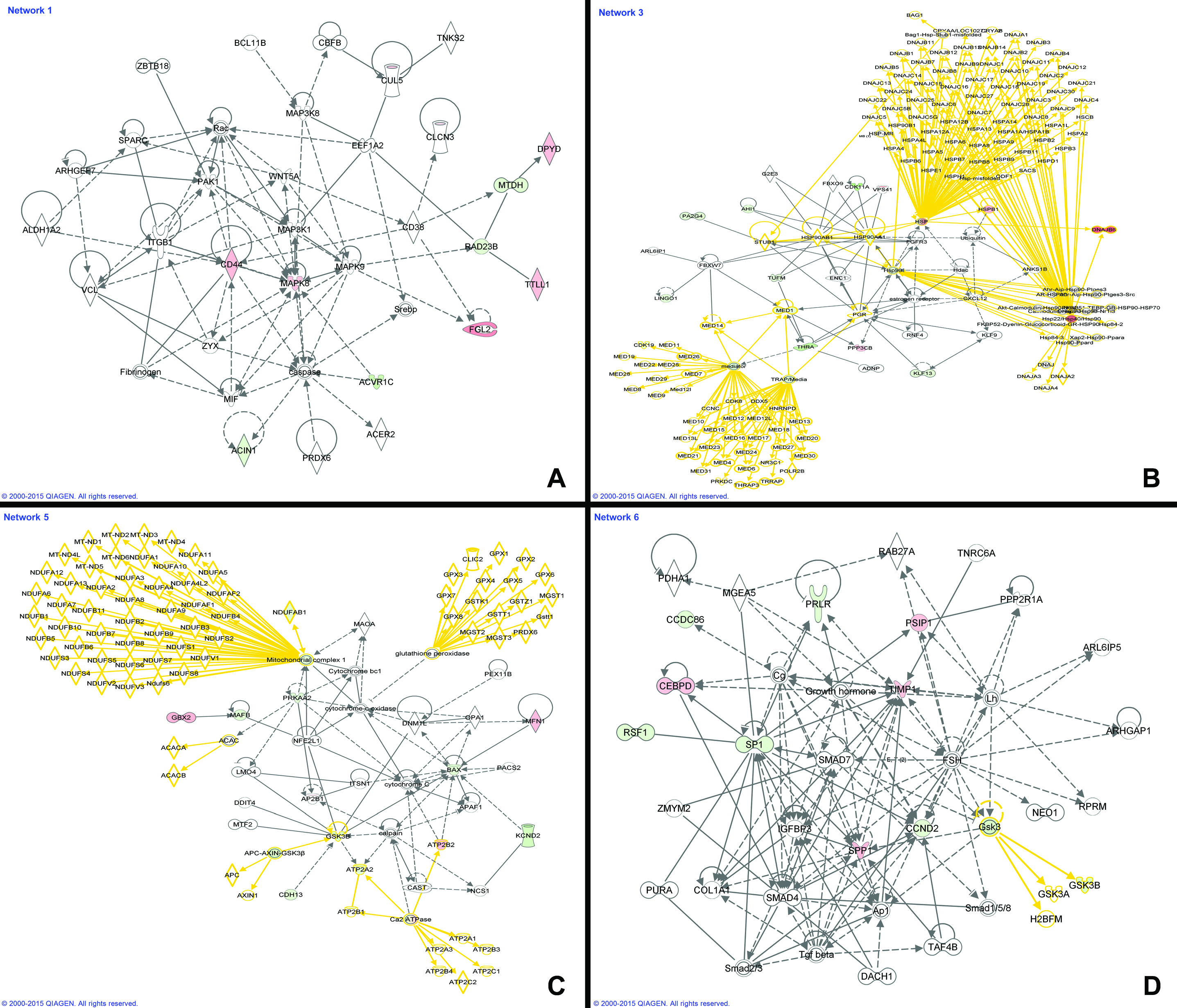

Supplement: Additional file 2: — Examples of TBI-C networks. TBI-C CD networks 1 (A), 3 (B), 5 (C), and 6 (D) (see Table 3) with all gene families, groups and complexes expanded to show the member genes and showing the relative expression values of potential GOI for TBI-C. red: relative increase in expression; green: relative decrease in expression; white: no change in expression; gold connections and outlines: expansion of gene families, groups and complexes in the original network. (TIF 4.30 mb) [file 12864_2016_2412_MOESM2_ESM.tif]

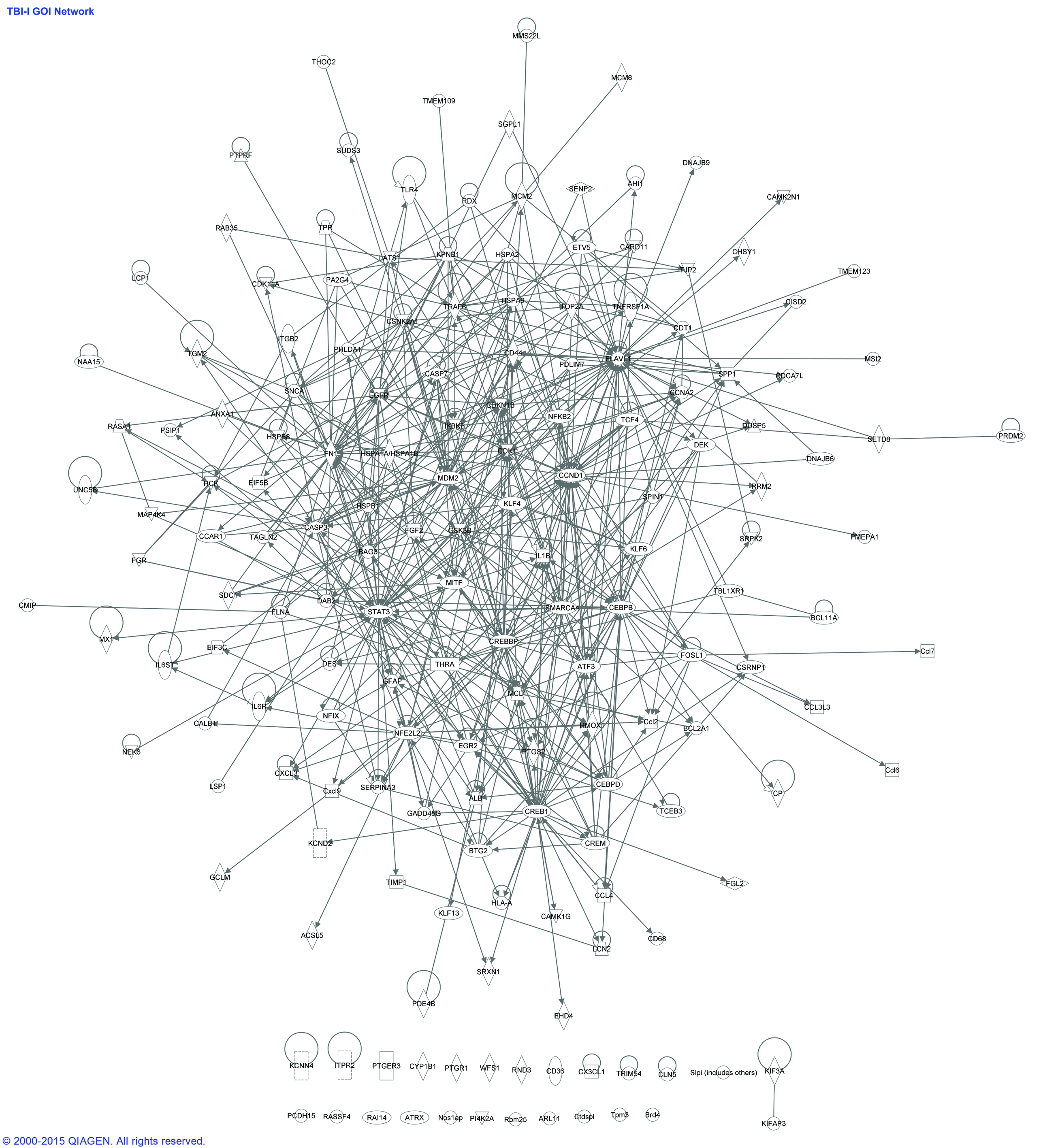

Supplement: Additional file 3: — The TBI-I GOI network. This is the resultant network when IPA connected our 170 TBI-I GOI using only direct (1st order) connections between the genes. 145 of the GOI formed an interconnected network, leaving 25 “orphan” genes. (TIF 4.20 mb) [file 12864_2016_2412_MOESM3_ESM.tif]

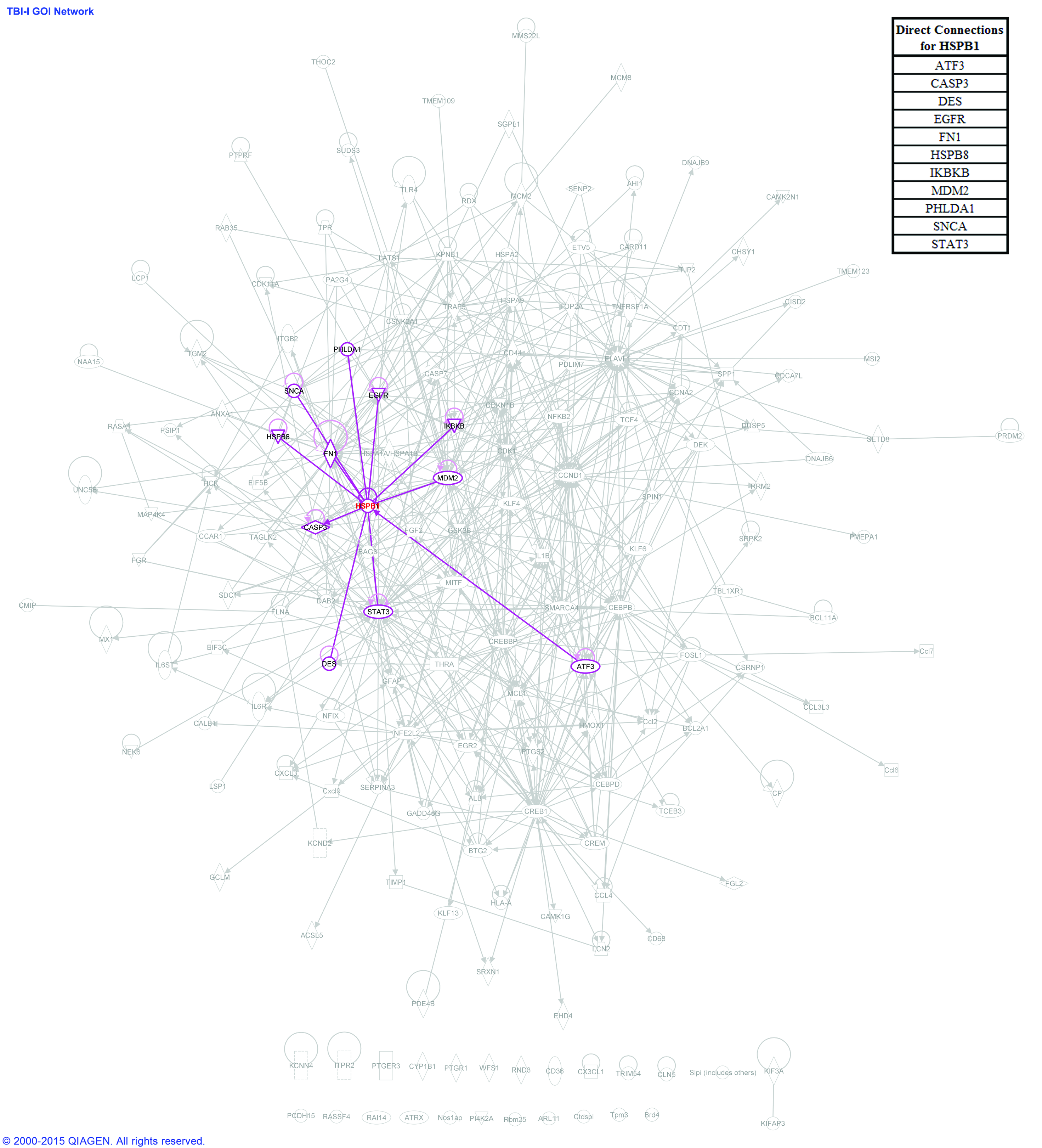

Supplement: Additional file 4: — An example of calculating the number of direct connections for the TBI-I GOI network. In IPA, the gene in question was selected (HSPB1 in this example). Then, its direct connections were selected by right clicking on HSPB1 and using the “select nearest neighbors” option (highlighted in purple). A list of the selected genes was exported and HSPB1 was removed from the list (upper right corner). The remaining genes were counted (11 in this example) and HSPB1 was ranked in the TBI-I gene interaction hierarchy (secondary tier) by this number. (TIF 3.99 mb) [file 12864_2016_2412_MOESM4_ESM.tif]

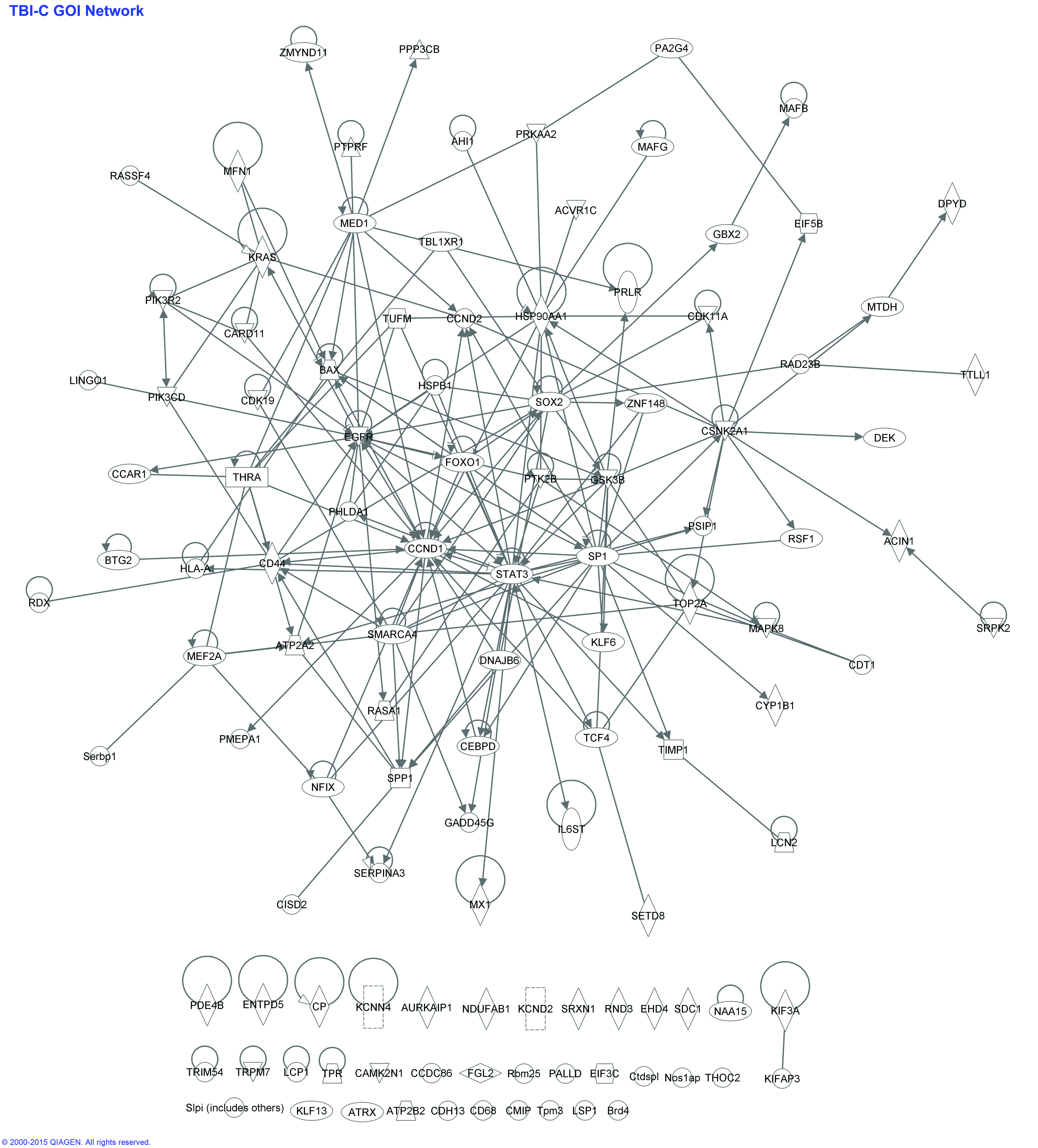

Supplement: Additional file 5: — The TBI-C GOI network. This is the resultant network when IPA connected our 115 TBI-C GOI using only direct (1st order) connections between the genes. 78 of the GOI formed an interconnected network, leaving 37 “orphan” genes. (TIF 4.84 mb) [file 12864_2016_2412_MOESM5_ESM.tif]

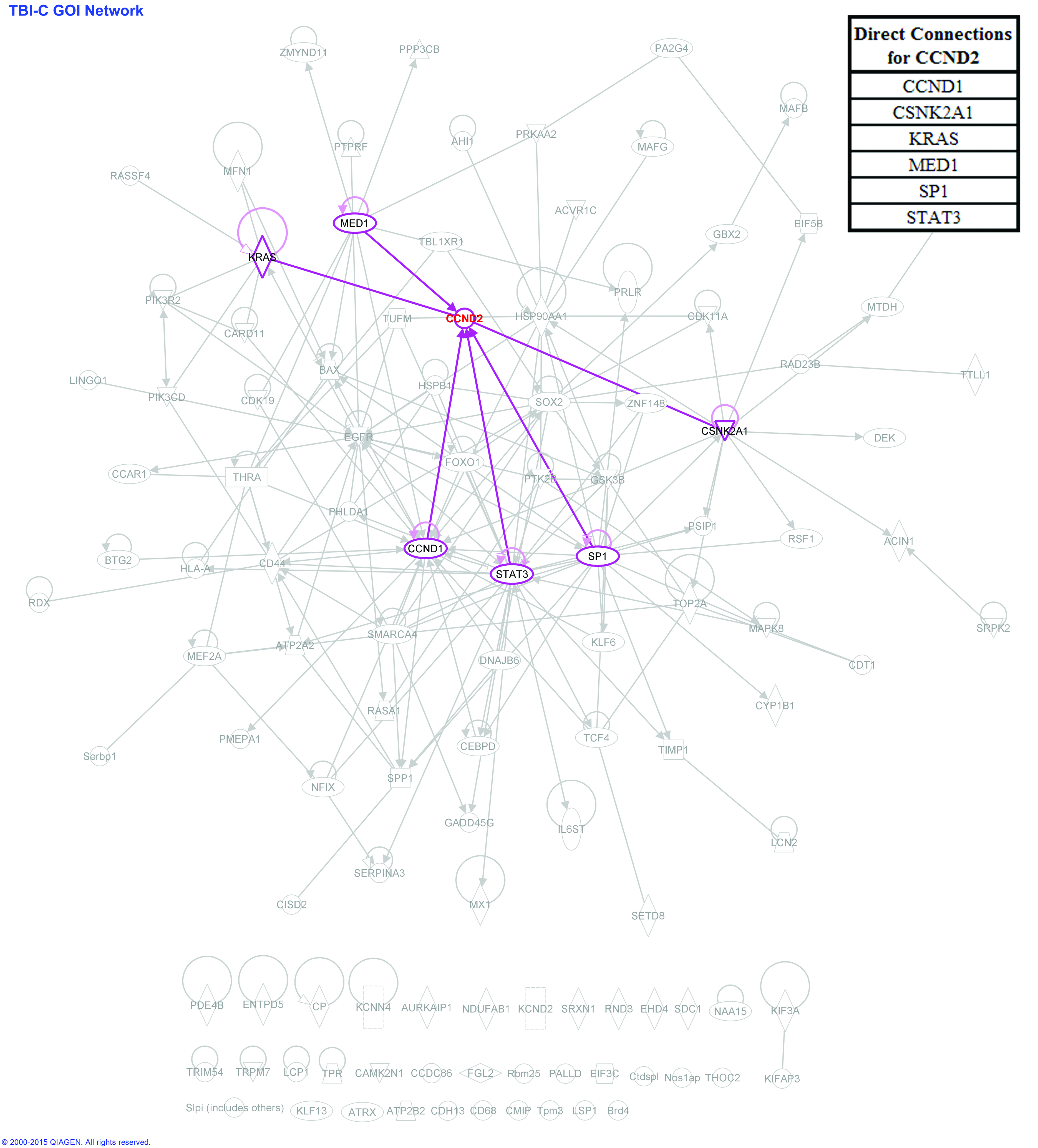

Supplement: Additional file 6: — An example of calculating the number of direct connections for the TBI-C GOI network. In IPA, the gene in question was selected (CCND2 in this example). Then, its direct connections were selected by right clicking on CCND2 and using the “select nearest neighbors” option (highlighted in purple). A list of the selected genes was exported and CCND2 was removed from the list (upper right corner). The remaining genes were counted (6 in this example) and CCND2 was ranked in the TBI-C gene interaction hierarchy (secondary tier) by this number. (TIF 4.69 mb) [file 12864_2016_2412_MOESM6_ESM.tif]
